# Compound Impacts of Fluvial Flooding and Sea-Level Rise on Benzo[*a*]pyrene Transport in the Lower Darby Creek Area Superfund Site, Pennsylvania, USA

**DOI:** 10.1021/acsestwater.4c00814

**Published:** 2025-06-20

**Authors:** Sean A. Woznicki, Joshua Barber, Jonathan B. Butcher, Jonathan Essoka, Maureen Harris, Megan Mehaffey, Bruce Pluta, Afshin Shabani, Pai Yei Whung

**Affiliations:** † Annis Water Resources Institute, Grand Valley State University, 740. W. Shoreline Dr., Muskegon, Michigan 49441, United States; ‡ Superfund Emergency Management Cleanup Division, Region 3, U.S. Environmental Protection Agency, Four Penn Center, 1600 John F. Kennedy Blvd., Philadelphia, Pennsylvania 19103-2029, United States; § Tetra Tech, Inc., 4000 Sancar Way Ste. 200, Durham, North Carolina 27709, United States; ∥ Center for Environmental Solutions and Emergency Response, Office of Research and Development, U.S. Environmental Protection Agency, Four Penn Center, 1600 John F. Kennedy Blvd., Philadelphia, Pennsylvania 19103-2029, United States; ⊥ AECOM, 5438 Wade Park Blvd, Raleigh, North Carolina 27607, United States; # Center for Public Health and Environmental Assessment, Office of Research and Development, U.S. Environmental Protection Agency, 109 T.W. Alexander Drive, Research Triangle Park, Durham, North Carolina 27709, United States; ∇ Center for Environmental Measurements and Modeling, Office of Research and Development, U.S. Environmental Protection Agency, 109 T.W. Alexander Drive, Research Triangle Park, Durham, North Carolina 27709, United States (retired)

**Keywords:** climate change, contaminated site, HEC-RAS, water quality, WASP

## Abstract

The compound effects of fluvial flooding, tidal dynamics, and sea-level rise (SLR) have the potential to mobilize pollutants at contaminated sites, which are often situated in flood-prone areas. We assessed the compound effects of these flood drivers on benzo­[*a*]­pyrene (B­[*a*]­P)-contaminated sediments in the Lower Darby Creek Area (LDCA) Superfund Site in Pennsylvania, USA. B­[*a*]­P, ubiquitous in the sediments of LDCA, is a known human carcinogen and is an indicator of polycyclic aromatic hydrocarbons in the environment. The LDCA is tidally influenced via the Delaware Bay, is projected to experience sea-level rise, and is situated within an active river floodplain. These conditions lead to potential B­[*a*]P transport within and out of the LDCA. Using a one-way coupling of the Hydrologic Engineering Center-River Analysis System (HEC-RAS) model and the Water Quality Analysis Simulation Program (WASP), we demonstrate that by 2050 fluvial flooding will continue to be the major driver of contaminant transport in the LDCA system. Fluvial-driven sediment transport defines B­[*a*]P deposition, which is largely influenced by tributary inputs and the distribution of B­[*a*]P in floodplain sediments. The complex patterns of B­[*a*]P redistribution at the LDCA, influenced by multiple drivers of flooding, demonstrate the utility of a coupled modeling approach to inform remediation and community resilience.

## Graphical abstract

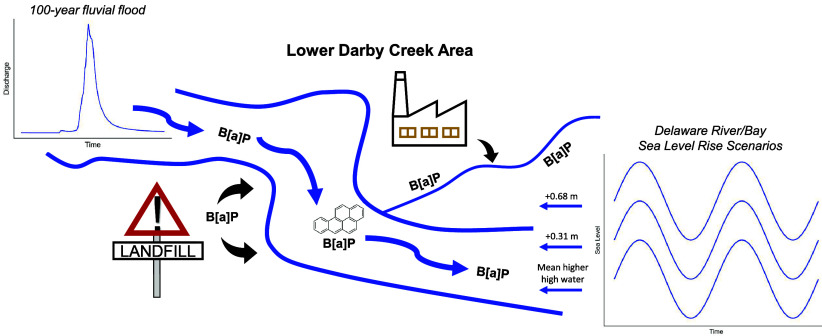

## 1 Introduction

As the intensity of extreme rainfall events increases, the occurrence of flooding also increases, leading to the mobilization and redistribution of contaminated floodplain soils and river sediments.^1^ Flooding of contaminated sites can release pollutants into floodwaters and transport them into surrounding environments, posing risks to human and ecosystem health.^2^ Many sites with legacy contamination listed in the Superfund National Priority List are situated next to rivers due to historical industrial and commercial development.^1^ For example, legacy and nonoperational landfills are often located in floodplains and coastal plains due to ease of access.^3^ These coastal sites are vulnerable to inundation from upland flooding, sea-level rise (SLR), tropical storms, and storm surge.^3,4^ This is a significant issue in the United States; the U.S. Government Accountability Office estimates that approximately 60% of all nonfederal Superfund National Priority List sites are in areas that may be impacted by fluvial flooding, SLR, and storm surge.^5^ Another analysis found that 327 Superfund sites are prone to climate-change-driven SLR or flooding, and 2 million people live within 1 mile of these sites.^6^

Previous studies have performed coastal-site and city-scale assessments of the mobilization, transport, and distribution of contaminants during extreme events and their aftermath, including tropical storms^7–11^ and fluvial floods.^12–14^ These studies typically use pre- and postevent sampling and spatial analysis to demonstrate the role of flooding and subsequent sediment transport in redistributing contaminants such as polycyclic aromatic hydrocarbons (PAHs) and heavy metals. For example, Dellapena et al.^7,8^ used pre- and postflood sampling and spatial analysis to quantify the mass of mercury-laden sediment delivered to Galveston Bay, TX, USA following Hurricane Harvey. Other sampling studies in coastal areas, such as New Orleans, LA, USA (Hurricane Katrina),^10^ New York City, USA (Hurricane Sandy),^11^ and North Carolina, USA (Hurricane Florence),^9^ concluded that redistribution of contaminants in soil and sediment occurred due to floodwaters. Fluvial floodplain studies have shown the potential of flooding to drive erosion and deposition of sediment sorbed with PAHs.^15,16^ Most hazard assessments focus on one driver at a time, rather than compound events that stack fluvial flooding, tides, and SLR, which can occur in coastal watersheds.^17^ Moftakhari et al.^17^ found that future sea-level rise exacerbates other flood drivers such as extreme high tides and fluvial flooding. However, the authors are unaware of any studies that model both the compounding effects of multiple flood drivers and the resultant contaminant transport. Site-scale analysis to develop projections of the compounding effects of these extreme events and future conditions at contaminated sites is critical to guide remediation actions and protect human health in adjacent communities.

We modeled the combined impacts of fluvial flooding, mean higher high water (MHHW) tide, and SLR on sediment and contaminant transport at legacy landfills in the Lower Darby Creek Area (LDCA) Superfund site^18^ in Darby Township and Folcroft Borough, Pennsylvania, USA (Figure 1). LDCA is prone to flooding and is situated among residential, commercial, and industrial land uses. The contaminated Operable Units (OUs) at LDCA (discrete areas for which assessment and response actions are associated), Clearview Landfill (OU1) and Folcroft Landfill (OU2), are situated on the Darby Creek floodplains, which are tidally influenced via the Delaware River and projected to be affected by SLR. Previous studies have demonstrated that SLR could compound extreme fluvial flooding in Darby Creek and its floodplain.^19^ Notably, OU1 resides next to the Eastwick residential neighborhood, which is within the 100 year floodplain. Waste disposal at the landfills ended in the 1970s, and although they are capped, the surrounding area contains legacy soil and sediment contamination, posing a threat to human and ecosystem health. Thus, flood-induced mobilization of contaminated sediments is a potential risk at the LDCA.

**Figure 1:**
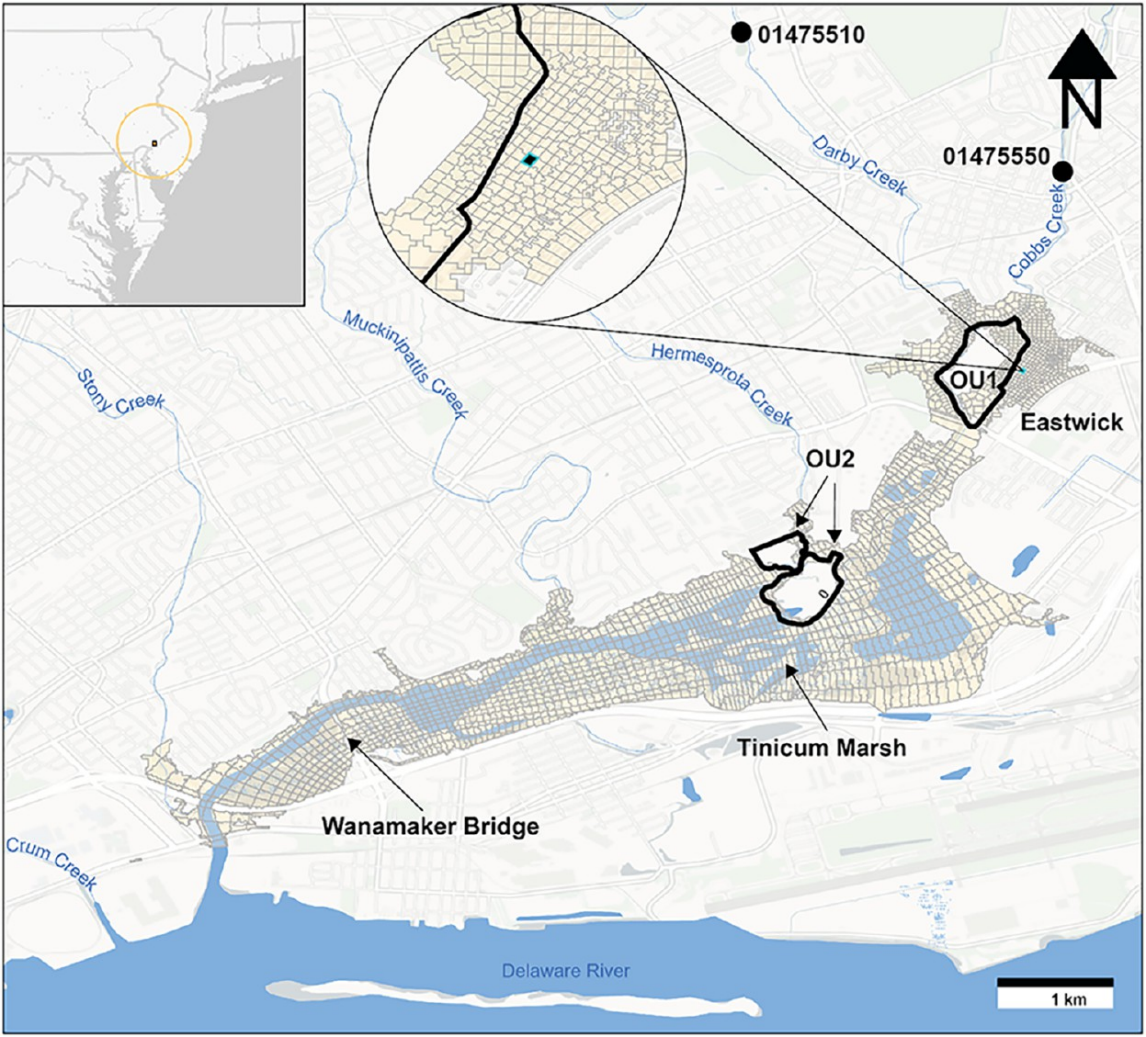
Location of the Lower Darby Creek Area, including OU1 and OU2, overlaid with the WASP (water quality model) grid. Detail in the Eastwick neighborhood (in circle) shows a water column and surface sediment cell examined in Section 3 (hereafter, “Eastwick Model Cell”).

Contaminants at the LDCA include various metals, PAHs, polychlorinated biphenyls, pesticides, and volatile organic compounds.^20,21^ In this study, we focus on the distribution, fate, and transport of benzo­[*a*]­pyrene (B­[*a*]­P), which is a highly hydrophobic, nonvolatile carcinogenic PAH.^22,23^ It is critical to understand the distribution of B­[*a*]P at contaminated sites because it is an indicator of total PAHs, many of which are carcinogenic.^24–26^ In aquatic systems, PAHs are typically adsorbed to sediment or suspended particulates, with transport driven by resuspension in the water column.^27^ Due to its hydrophobic nature, B­[*a*]P is present in high concentrations in aquatic sediments and soil compared to surface waters.^28^ Aquatic sediment samples near LDCA contained elevated PAH concentrations more often and at much higher levels than other contaminants, often an order of magnitude greater than background concentrations.^20^

We coupled the Hydrologic Engineering Center-River Analysis System (HEC-RAS) model and the Water Quality Analysis Simulation Program (WASP) to simulate B­[*a*]P transport under fluvial flooding, tidal fluctuations, and sea-level rise conditions. HEC-RAS is a widely used hydraulic model, while WASP has been used for more than 50 years^29^ for a wide variety of systems and chemicals, including PAHs,^30^ polychlorinated biphenyls,^31^ heavy metals,^14,32–36^ and naphthenic acids.^37^ We developed one-way coupling of these models to simulate the impacts of compound flooding events on contaminated sites. Approaches such as these are critical because climate change and extreme weather can alter contaminant fate and transport and can negatively affect a site’s original remediation design and long-term management.^38^ Site-specific projections of future flooding, sea level, and contaminant fate and transport, as conducted here, are necessary for adaptation planning, management, and long-term operations at the 300-plus flood- and SLR-prone Superfund sites across the United States.

## 2 Materials and Methods

### 2.1 Study Area and Contamination History

The LDCA Superfund site is situated along Darby Creek and is tidally influenced via the Delaware River (Figure 1). It is in a highly urbanized and industrial setting and borders multiple residential areas. The sites of the study encompassed two landfill OUs, discrete areas for which assessment and response actions are associated: OU1 includes Clearview Landfill, the Eastwick Regional Park, and a portion of the Eastwick neighborhood (single and multifamily housing) and OU2 consists of the Folcroft Landfill and Folcroft Annex and borders the John Heinz National Wildlife Refuge at Tinicum, a freshwater tidal marsh (hereafter, “Tinicum Marsh”).^18^ Contamination at OU1 and OU2 is due to the historic and largely undocumented disposal of municipal and industrial wastes, leading to their placement on the Superfund National Priorities List in 2001.^18^

Clearview Landfill was operated from the 1950s to 1973 when it was ordered to cease waste disposal operations by the state of Pennsylvania, although unpermitted hazardous waste transfer, storage, and disposal continued into the 1980s.^20^ Cleanup at Clearview Landfill began in 2019 and is ongoing, with most work to be completed in 2024.^18^ The landfill is now capped, and the highest point is at a greater elevation (>24 m above mean sea level) than the 500 year floodplain elevation (5.3 m above mean sea level).

Folcroft Landfill and Annex was operated from approximately 1961 to 1974, accepting municipal, demolition, industrial, and commercial wastes as well as incinerator ash and sewage sludge.^21^ Folcroft Landfill was capped with a 2 ft soil cover in 1977.^21^ The Feasibility Study evaluating potential long-term cleanup options is ongoing and expected to be approved in 2024.^18^ Other sources of contamination include nearby industrial operations, crude oil storage, the former Delaware County Sewage Treatment Plant sludge disposal, and the former Delaware County Incinerator.^20^

Aquatic sediment samples in Darby and Cobbs Creeks near LDCA contained elevated PAH concentrations more often and at much higher levels than other contaminants, up to 6.3 mg kg^–1^ in Darby Creek sediments adjacent to Clearview Landfill.^20^ This exceeds background B­[*a*]P concentrations in surface soils by an order of magnitude, with soils (depth: 0–15 cm) measured at a nearby residential neighborhood (mean, 0.14 mg kg^–1^; range, 0.05–0.49 mg kg^–1^; *n* = 7) and Tinicum Marsh (mean, 0.21 mg kg^–1^; range, 0–0.38 mg kg^–1^; *n* = 4).^20^ This is also an order of magnitude greater than mean background B­[*a*]P concentrations of 0.7 mg kg^–1^ from a separate study conducted across diverse locations in Eastern Pennsylvania.^39^

Contaminated soils and sediments are widespread within the Darby Creek floodplain, making the LDCA vulnerable to extreme flooding events. In the future, the study area may also experience SLR-driven inundation^40^ due to its connection to the Delaware River and the Delaware Bay. Because B­[*a*]P is a broad indicator of total PAH burden, understanding the distribution, fate, and transport of B­[*a*]P during flood events allows for a comprehensive view of PAHs and contaminants with similar characteristics at the LDCA.

### 2.2 Modeling Framework

We used HEC-RAS 2D (v 5.07 ^41^) to simulate the hydrodynamics of the 100 year flood, MHHW, and SLR. The HEC-RAS output was linked to the Water Quality Analysis Simulation Program (WASP8 v 8.32 ^42^) with Advanced Toxicant Module^43^ to simulate sediment and B­[*a*]P fate, mobilization (erosion and resuspension), transport, and deposition.

To quantify sediment and B­[*a*]P transport in LDCA, we used four HEC-RAS-WASP simulations: (1) 100 year fluvial flood event with fixed downstream tidal elevation, a baseline simulation; (2) 100 year flood simulation reflecting tidal signal; and (3 and 4) 100 year flood simulation reflecting tidal signal coupled with two future relative sea-level (RSL) rise scenarios that correspond with future global mean sea-level (GMSL) rise: intermediate-low and intermediate-high scenarios.

The modeling domain extended from north of the confluence of Darby Creek and Cobbs Creek (upstream of OU1 and OU2), along the Darby Creek floodplain downstream, to the confluence of Darby Creek and the Delaware River, covering approximately 9.5 km of Darby Creek (Figure 1). This domain was defined to necessitate separate flood hydrographs from both creeks while not extending too far from OU1 to limit computational grid size (and time) and to include the areas of B­[*a*]P contamination in these creeks.

### 2.3 Hydrodynamic Model

#### 2.3.1 HEC-RAS Grid

A combined one-dimensional/two-dimensional (1D/2D) HEC-RAS was implemented using unsteady flow simulations to produce depth and velocity outputs for a flood event corresponding to the 100 year return period, 24 h precipitation event, with a computation time step of 1 s. The HEC-RAS terrain model and 2D mesh were created using a combination of 2015 lidar point data and a derived bare earth 1 m digital elevation model^44^ plus 2012 river channels bathymetric survey data.^45^ The entire model domain was configured as a 2D HEC-RAS model, except for three bridges, whose decks may become submerged during flooding, which were represented as short 1D HEC-RAS links. Two other bridges in the study area were unlikely to be overtopped and thus were configured into the 2D terrain and mesh using their pier, embankment, and abutment geometries obtained from as-builts. The final grid consisted of three 2D flow areas directly connected by 1D reaches for bridges, with 67,089 grid cells. Manning roughness coefficients were assigned based on land cover classified from high-resolution ortho-imagery and LiDAR using coefficients based on Kalyanapu et al.^46^ and adjusted to reflect site-specific conditions during calibration against the Federal Emergency Management Agency (FEMA) 100 year flood extent.

#### 2.3.2 HEC-RAS Boundary Conditions

Landside flood hydrographs for the 100 year, 24 h design storm were generated using the HEC Hydrologic Modeling System (HEC-HMS, v4.3) for a 72 h period calibrated to USGS gages 01475510 (Darby Creek near Darby, PA) and 01475550 (Cobbs Creek at Darby, PA). HEC-HMS produced hydrographs for Darby Creek and Cobbs Creek at the upstream model boundary and all Darby Creek tributary outlets: Muckinipattis Creek, Hermesprota Creek, Stony Creek, and unnamed minor tributaries (Figure 1). National Oceanic and Atmospheric Administration (NOAA) Atlas 14 ^47^ precipitation intensity duration estimates for the design storm were obtained from the NOAA Precipitation Frequency Data Server^48^ and used to create HEC-HMS hourly rainfall input data at 5 min intervals using the frequency storm method. The 100 year flood stage of Darby Creek, just downstream of its confluence with Cobbs Creek, is 6.35 m, timed with the peak MHHW tidal condition, while the preflood stage at that same location is 1 m.

Tidal boundary conditions were implemented using NOAA tide stations^49^ at Philadelphia, PA (station no. 8545240; coordinates: 39° 56.0 N, 75° 8.5 W) and Billingsport, NJ (station no. 8538552; coordinates: 39° 51.0 N, 75° 15.0 W). The Philadelphia station produces full tidal time series, datums, predictions, and harmonic constituents, while the Billingsport, NJ, station, closest to the downstream model boundary, provides subordinate tide predictions only. NOAA references Billingsport station tides to the Philadelphia station tides based on the following additive time offsets (high tide: −35 min; low tide: −28 min) and multiplicative height offsets (high tide: 0.93, low tide: 0.95). These offsets were applied to the Philadelphia tide record to develop representative tidal boundary conditions at the confluence of Darby Creek and the Delaware River. The tidal cycle was approximated using a sine function between the low and high tide values to generate a subhourly tide cycle time series with 5 min time steps to create a dynamic boundary condition. The MHHW tidal datum corresponding to tidal station no. 8545240 (Philadelphia, PA) of 0.97 m NAVD88 was used and synchronized such that MHHW was temporally aligned to the storm hydrograph peak. Further details on tidal calibration are in the Supporting Information. The static, nontime varying downstream boundary condition experiment was set to 0.8 m, representing mean high water.

Future SLR scenarios were derived from NOAA GMSL- and RSL-rise scenarios^50^ and imposed as boundary conditions. We used the RSL-rise estimates for 2050 based on the intermediate-low (+0.5 m) and intermediate-high (+1.5 m) GMSL rise by 2100 scenarios.^50^ Further, our analysis uses the 50th percentile subscenario within those GMSL rise scenarios, provided for the Philadelphia, PA tide station (station no. 8545240). The RSL projections for 2050 at this tide gage are +0.31 m (intermediate-low) and +0.68 m (intermediate-high), which were applied uniformly to the tidal boundary condition time series for LDCA. The RSL +0.31 m increases the preflood stage at the confluence of Darby Creek and Cobbs Creek to 1.28 m (from 1.01 m), while the RSL +0.68 m increases the stage to 1.63 m. Both scenarios have 6.26 m stages at that location during the peak of the 100 year flood.

Although sea-level rise is projected to change tidal characteristics globally, we did not account for these changes. Pickering et al.^51^ found that +0.5 m SLR would decrease Philadelphia’s mean high water (MHW) tide by 0.01 m, and the tidal range would contract by 0.06 m. Under greater SLR (+2 m), MWH would decrease by 0.05 m and the range would decrease by 0.16 m. Based on these estimates of minor tidal change in response to SLR in Philadelphia, we are confident in our approach to setting tidal and SLR boundary conditions.

### 2.4 Hydrodynamic Water Quality Model Linkage

Two types of information must be transferred from HEC-RAS to WASP: (1) static information regarding the grid, grid cell geometric properties, and the relationships between the HEC-RAS and WASP grids; and (2) unsteady time series information on flows, velocities, and volumes.^14^ WASP was configured with a grid size larger than that of HEC-RAS, which allows a longer time step and more manageable run times in WASP. HEC-RAS grid cells were assigned to WASP grid cells based on the location of the HEC-RAS grid cell centroid relative to the WASP index grid. HEC-RAS face flows that correspond to boundaries between WASP grid cells were identified by filtering the full set of HEC-RAS faces to retain those that join HEC-RAS grid cells belonging to different WASP grid cells. Hydraulic information from HEC-RAS, aggregated to WASP cells, was passed from HEC-RAS to WASP at 5 min intervals. Full details of the linkage process are in the Supporting Information.

### 2.5 Water Quality Model

#### 2.5.1 WASP Model Description

WASP8 is a spatially resolved differential mass balance fate and transport model that solves the advection–diffusion equation.^29^ The mass balance equation is integrated over a completely mixed finite volume/segment, where the segments can be configured in arbitrary arrangements to form one-, two-, or three-dimensional networks.^29^ The equation is solved for each segment and time step with the forward Euler numerical approximation with varying time steps.^43^ Relevant model routines are described below, with additional parametrization information in the Supporting Information.

Mechanistic solids transport was implemented using the van Rijn equations,^52^ which calculate dynamic settling, deposition, erosion, and resuspension velocities (solids option 1 in WASP8). The mechanistic transport routines allow for the simulation of erosion during high flows and deposition during slower flows.^43^ Settling is calculated based on particle size and density, while deposition, erosion, and resuspension are functions of bottom shear stress.^53^ Further, erosion and resuspension are dictated by whether the behavior of the sediment bed is cohesive or noncohesive.^53^ Ultimately, these processes are a function of particle characteristics (density and diameter), solid constants, and surface water flow velocity.^53^ WASP8 conducts a solid mass balance for each sediment segment and time step. Using the Dynamic option, the sediment segment has a constant bulk density that allows for volume to change with erosion or deposition.^43^ Full details of the mechanistic solids transport routines are presented in Ambrose et al.,^53^ and a summary is provided in Knightes et al.^43^

Particle attachment, or interaction (sorption and desorption) between solid particles and toxicant chemicals, is a significant process in the fate and transport of contaminants in surface waters due to the ubiquity of solids in those waters. This is particularly true for hydrophobic contaminants such as B­[*a*]­P. We used the equilibrium partitioning routines in WASP8 (which were also included in previous versions of WASP).^43,53^ Equilibrium partitioning assumes that the sorption reactions are fast relative to environmental processes (and time steps simulated), allowing for the assumption of equilibrium.^53^ For environmentally relevant concentrations, equilibrium sorption can be represented as in eq 1 ^54^

1 $$C_{i}^{S}=K_{d,i}C_{i}^{W}$$

where, for chemical *i*, *C*_*i*_^*S*^ is the chemical concentration in the solid phase (mg L^–1^), *C*_*i*_^*W*^ is the chemical concentration in the aqueous phase (mg L^–1^), and *K*_*d,i*_ is the partition coefficient (L kg^–1^). This is solved simultaneously when multiple solid classes are present.

Model verification has been performed for the solids transport and particle attachment algorithms by comparing them with analytical solutions.^53^

#### 2.5.2 WASP Grid

The WASP grid (Figure 1) was developed in two parts. For OU1 and upstream, the HEC-RAS interface was used to generate a coarser WASP model grid (mean grid cell size of 2,637 m^2^), with HEC-RAS grid cell centroids allocated to WASP grid cells. To align HEC-RAS and WASP grids in the OU2 area, we generated a curvilinear-orthogonal index grid at the desired coarser spatial resolution and assigned HEC-RAS grid cells to that grid. The curvilinear-orthogonal grid was generated using the Visual EFDC software package.^55^

The WASP grid contains grid cells that transition between wet and dry (or are always dry) during some flood simulations. However, WASP will crash when the grid cell water volume is equal to zero, and the simulation will also slow drastically as volume approaches zero. In order to address this issue, the code for creating the WASP hydrodynamic linkage in the “.hyd” file specified additional condition for the grid cell when it is simulated as almost dry: (1) set a grid cell’s minimum volume to 4 m^3^, (2) set the depth as that volume divided by area or 0.01 m, whichever is greater, and (3) set velocity to 0 m s^–1^ unit within the grid cell. We configured the water column as a two-dimensional lateral grid, with each grid cell underlain by a surface sediment layer.

#### 2.5.3 Sediment and Contaminant Sampling Data

Surface sediment B­[*a*]P concentrations and sediment physical characteristics (i.e., grain size) from the creek sediments, floodplain and upland soils, and Tinicum Marsh in the LDCA were collected over multiple sampling campaigns between 2002 and 2012.^20,21,45,56^ No single campaign provided a comprehensive picture of the distribution of contaminants in Lower Darby Creek and its floodplains. Therefore, the sampling results from all campaigns were used to establish the model’s initial conditions. Locations of all samples from the campaigns detailed below are presented in the Supporting Information.

The 2002–2007 sampling campaign collected sediment samples at 38 locations spanning Darby Creek, Cobbs Creek, OU1, and Tinicum Marsh.^20^ Samples were collected using large stainless steel precleaned spoons in wadable areas and using a Ponar sediment sampler when boat access was required.^20^ Soil samples were collected between 2003 and 2006 in and near OU1 and the Eastwick neighborhood and in 2007 in the wildlife refuge.^20^ A GeoProbe drill rig with hydraulically powered hammer drove a MacroCore tube assembly with a 1.5 in. diameter sampler to the target depth.^20^ Sample depths were surface (0–6 in.), medium (6–10 in.), and deep (10–20 in.) depending on the location.^20^ A separate campaign at OU2 in 2006 collected 40 surface soil samples (0–6 in.) and 16 subsurface soil samples (16–24 in.) across 53 soil borings using a GeoProbe drill rig.^21^

The 2012 sampling campaign measured contaminant distributions and sediment physical characteristics after flooding from Hurricane Irene in 2011 (which made landfall as a tropical storm) based on 35 operationally defined Decision Units (DUs), primarily in OU2 and Tinicum Marsh.^45,56^ The DUs were delineated to represent areas with uniform sediments, habitat conditions, and mobilization, transport, and fate processes.^45^ Within each DU, 50 surface sediment samples (0–7.6 cm depth or 0–3 in.) were collected from a 7/8 in. diameter tube on a randomized grid and composited to produce a representative sample for each DU, which was used for physical and chemical characterization.^45^

#### 2.5.4 WASP Benzo­[*a*]­pyrene Parametrization

The resulting B­[*a*]P sediment concentration maps combine areas that were sampled before and after the 2011 flood, which was necessary for complete coverage of the model domain. That the different samples are not all coincident in time presents a potential source of uncertainty, as it is likely that their redistribution has occurred over time. We used the B­[*a*]P results from the DU composites directly, where available, to specify the initial conditions for B­[*a*]P concentrations in surface sediments (Figure 2). This helped preserve the correlation between the sediment type and B­[*a*]P concentration and established post-Hurricane Irene (2011) conditions. The remainder of the B­[*a*]P concentrations from point measurements (pre-2011) were interpolated using inverse distance weighting (output grid cell size = 22.24 m, power = 2, variable search radius with six points) and area-weighted to the WASP grid (Figure 2).^57^ For use in WASP, B­[*a*]P concentrations in mg kg^–1^ were converted to volumetric units (mg L^–1^) by multiplying B­[*a*]P (mg kg^–1^) with the sum of the volumetric sediment concentration and φ *K*_d_^–1^, where *K*_d_ is the partition coefficient and φ is the porosity, to account for the dissolved fraction present in porewater. For B­[*a*]­P, the dissolved fraction in the porewater is an insignificant proportion of the total mass. The B­[*a*]P *K*_d_ used was 15,000 L kg^–1^, a typical value for partitioning of B­[*a*]P to environmental sediments with organic carbon content of around 2%;^58^ organic carbon fractions are in the range of 1.5–5% in LDCA.

**Figure 2:**
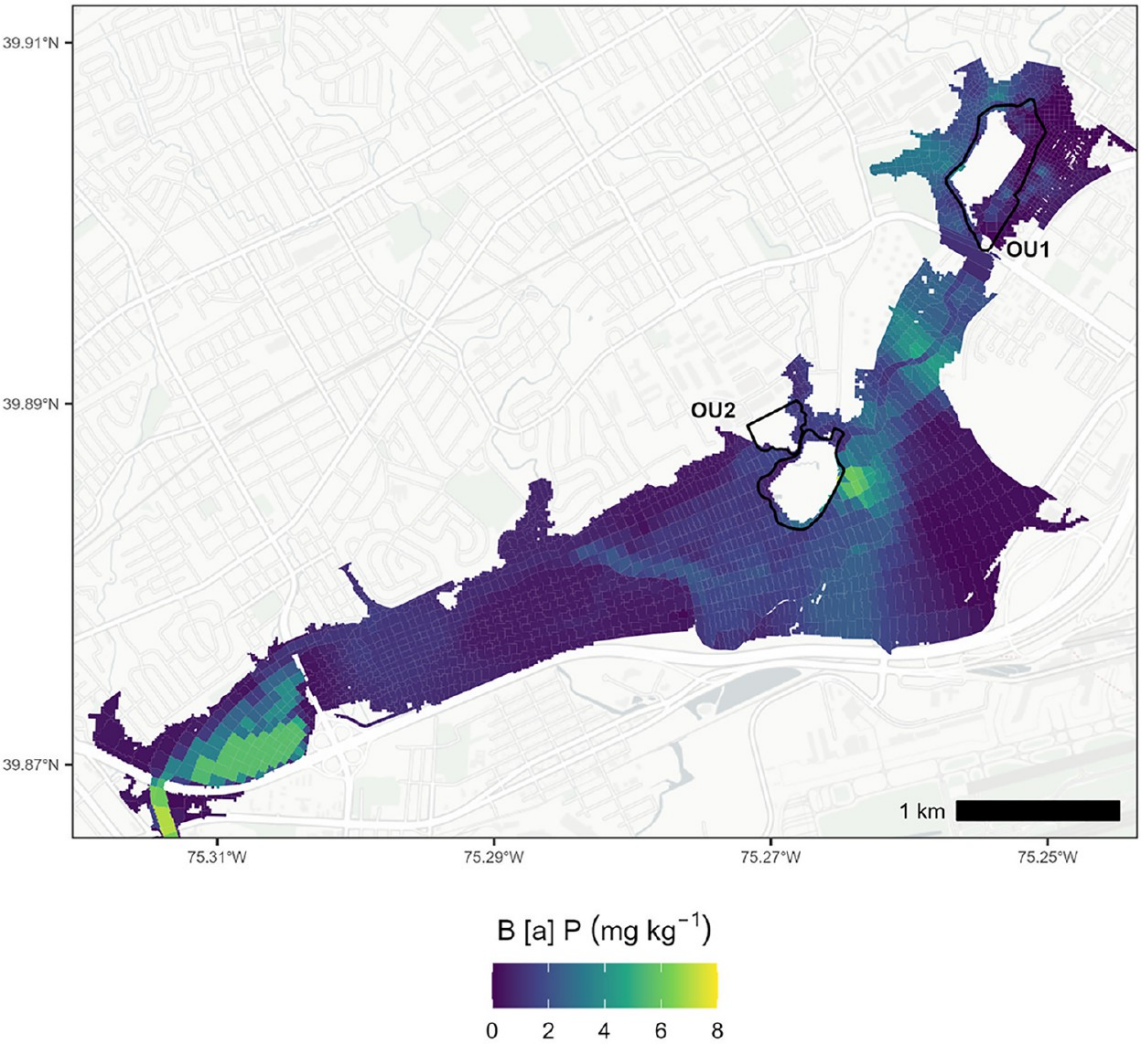
Initial benzo­[*a*]­pyrene concentrations (mg kg^–1^) in the sediment and soil for the WASP model, parametrized using point measurements and Decision Unit composite data.

#### 2.5.5 WASP Sediment Parametrization

The process-based sediment simulation with the dynamic sediment bed option was used to simulate changes in the benthic cell volume. Sand, silt, and clay fractions are represented. Sediment characteristics were defined using the previously discussed point data, DU composite data, and additional USDA soil survey data.^59^ These data were used to establish the general distribution of the sediment classes. The sand fraction dominates the main channel, while the marsh area is predominantly fine sediment.

The WASP model specifies sediment concentrations on a volumetric basis. The initial volume of each benthic cell is equal to the cell area multiplied by the assumed sediment depth, while the volume of sediment in each grid cell is equal to the total volume of the benthic cell, corrected for porosity. The surface benthic layer is active—accumulation of volume and depth occurs with deposition, and erosion causes loss of volume and depth.^60^ Surface benthic grid cells in the WASP were set to a fixed depth of 0.91 m. This depth was selected through trial and error to be sufficient to prevent grid cells from being entirely scoured of sediment during simulated flood events while not assuming an unlimited supply of scourable material. For the “dynamic sediment bed option” used with the ″process-based sediment simulation”, WASP preserves the initial bulk density and porosity of sediment segments while changing the volume of sediment in response to fluxes of different size classes. Initial values for parameters describing the bulk density, porosity, settling, resuspension, erosion, and deposition are listed in the Supporting Information.

#### 2.5.6 WASP Boundary Conditions

The boundary conditions for tributary inflows of B­[*a*]P (in surface sediment and suspended sediment) during storm events are uncertain due to limited suspended sediment boundary condition data. Surface water and suspended sediment samples at ten locations in both Darby Creek and Cobbs Creek, along with numerous samples adjacent to and downstream of the landfill OUs, were collected as a one-time sampling event in 2002.^20^ The assumption for primary transport of B­[*a*]P is that it is with sediment due to its low solubility in water. This suggests that additional contaminated sediment is likely to be transported into the study area during flood events. The B­[*a*]P boundary conditions in Darby Creek (1.4 mg kg^–1^) and Cobbs Creek (3.0 mg kg^–1^) were specified based on the means of measured B­[*a*]P concentrations sorbed to sediments (ten samples from each tributary) multiplied by the assumed suspended sediment concentration (50 mg L^–1^, obtained from limited monitoring data). For Hermesprota and Muckinipattis Creeks, the B­[*a*]P boundary concentrations on influent suspended sediment were assumed to be the same as those obtained in the DU composite samples for Hermesprota Creek (1.2 mg kg^–1^).^56^ Other minor tributary concentrations (Stony Creek) were set equal to the concentrations for surface sediment reported in the urban background samples (0.14 mg kg^–1^) near the site.^20^

#### 2.5.7 WASP Sensitivity Analysis

Because of the lack of water quality measurements during storm events to use as calibration data, sensitivity analyses are the major source of insight into uncertainty in model predictions for Lower Darby Creek. The 100 year flood baseline simulation (without tidal fluctuation and future SLR) was used for all sensitivity runs. We used one-at-a-time sensitivity analyses to quantify the effects of changes in model parameters related to sediment and contaminant scour, deposition and transport, model boundary conditions, and model initial conditions on simulated changes in the surface sediment contaminant concentration from pre- to postflood. Each parameter was increased and decreased within expected reasonable ranges (using the specified default ranges) to examine relative changes in outputs per change in inputs. We also included a test of sensitivity to peak discharge by comparing the result for the 10 and 500 year flood events to those from the 100 year event, with the discharge indexed at the 1D connection at the bridge between the upstream and downstream of the study area. We summarized the sensitivity analysis from 15 simulations using the complete wetted model grid and the portion of the grid that encompasses the Eastwick neighborhood near OU1. Parameter sensitivity coefficients are defined as the fractional change in the WASP output (e.g., B­[*a*]P concentration in sediment, average across the WASP grid) per unit change in the parameter value. The sensitivity coefficient (*S*) is presented in eq 2, where *C* is the average B­[*a*]P concentration of B­[*a*]P in surface sediment at the conclusion of the simulation and *x* is the input parameter value. Subscripts 0 and 1 represent the base simulation and the sensitivity simulation, respectively.

2 $$S=\frac{\frac{c_{1}-c_{0}}{c_{0}}}{\frac{x_{1}-x_{0}}{x_{0}}}$$

Sensitivity coefficients were calculated for positive and negative perturbations of each parameter because the response may be nonlinear. In the case of negative perturbations (reducing the parameter value), *S* is multiplied by −1 to aid in interpretation: when the sign of the perturbation and the sign of the response match, there is a positive correlation between the parameter and the result. In this manner, a negative perturbation with a positive *S* would indicate that reducing that parameter value increases the B­[*a*]P concentration.

## 3 Results and Discussion

### 3.1 Flood Depth and Extent

Maximum flood depth and extent over the simulated event are driven by the 100 year flood from Darby and Cobbs Creek, with relatively small influence from SLR (Figure 3). As stated previously, the 100 year fluvial flood stage at the confluence of Darby Creek and Cobbs Creek is 6.35 m, increasing from 1 m prior to the onset of the flood. With the addition of SLR, the maximum flood depth in the system increases by 0.04 m with RSL +0.31 m and by 0.13 m with RSL +0.68 m, with effects of SLR reaching just upstream of OU2. However, in individual model cells, the effect is more pronounced. Near the confluence with the Delaware River, the increase can be higher than the SLR, but this effect is mostly confined to the channel and Tinicum Marsh, while slight increases or decreases in flood depth (<0.1 m) occur further upstream near OU1 and Eastwick. This spatial trend is similar to that of a previous compound flooding study at Darby Creek, which noted that the effect of SLR is most prominent downstream and near Tinicum Marsh.^19^ The RSL +0.68 scenario produced the greatest changes from the static tide scenario: a mean of +0.2 m across all model cells (maximum increase = +1.22 m, maximum decrease = −0.34 m). A full summary of these changes is presented in Table S2 (comparison with the static downstream boundary) and Table S3 (MHHW tidal boundary) of the Supporting Information.

**Figure 3:**
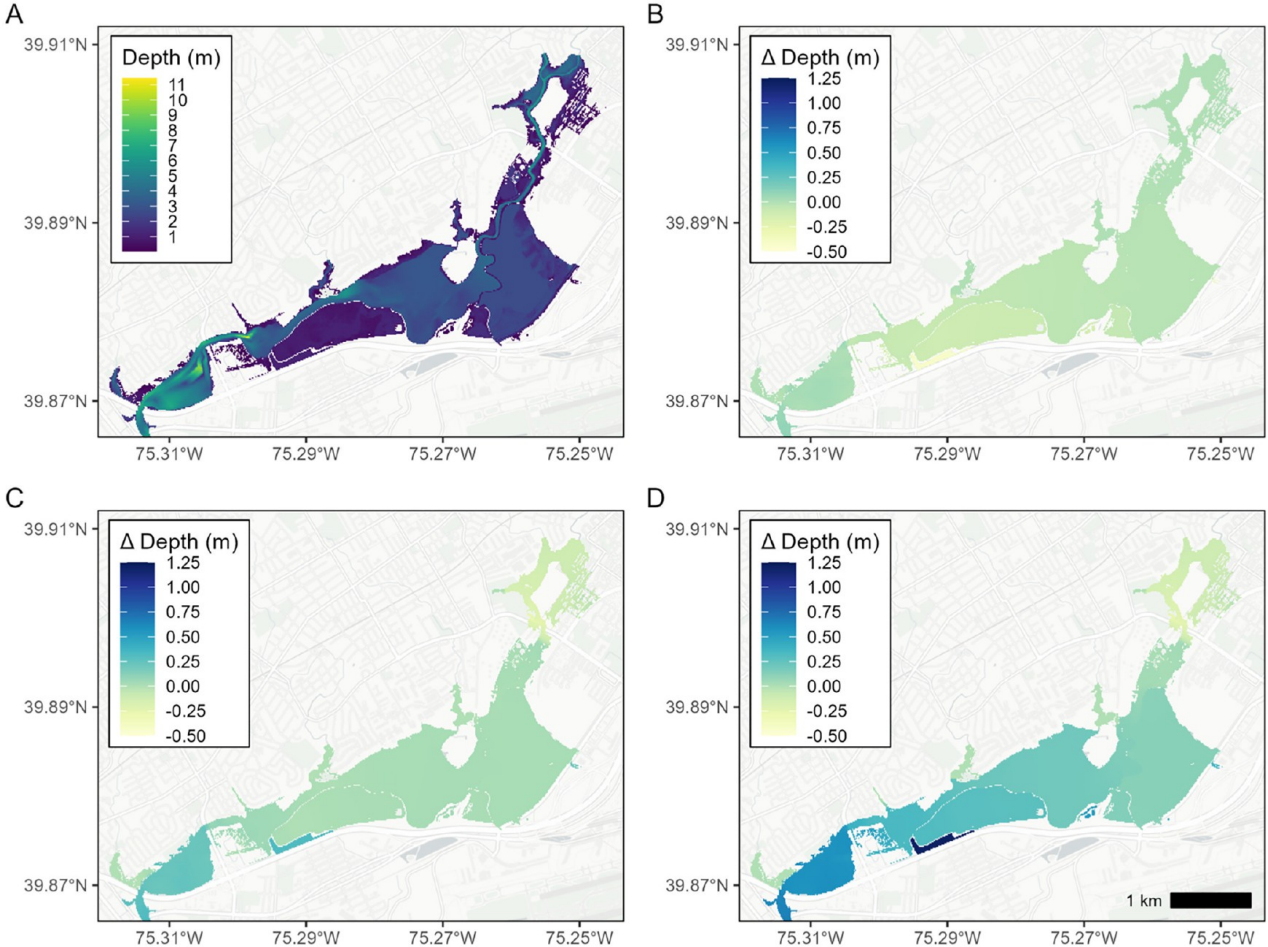
(a) Maximum flood depth for a 100 year flood with static tidal boundary; (b) difference in flood depth: 100 year flood with MHHW minus 100 year flood with static tidal boundary; (c) difference in flood depth: 100 year flood with +0.31 RSL minus 100 year flood with static tidal boundary; and (d) difference in flood depth: 100 year flood with +0.68 RSL minus 100 year flood with static tidal boundary.

### 3.2 Sensitivity Analysis

The baseline simulation resulted in a 0.61% decrease in B­[*a*]­P concentration in surface sediment across the entire grid (−1.29% in the Eastwick neighborhood). Sensitivity analysis results should be compared to these baseline decreases (Table 1). Changes in model parameters and boundary inflow concentrations produced a relatively small variability in the final surface sediment concentrations, with average concentrations in the whole grid ranging from −0.08 to −1.7% and from −0.98 to −2.8% within the Eastwick neighborhood (Table 1). The overall decrease in average B­[*a*]P concentrations sorbed to sediment, regardless of parameter change, could be explained by a dilution by a lower concentration of B­[*a*]P in sediments from tributaries deposited during flood events. However, some individual grid cells with low B­[*a*]P concentrations in the Tinicum Marsh and east of OU1 showed increases under all scenarios. Figures S3–S10 in the Supporting Information show the spatial patterns and distribution of the parameter sensitivity across the WASP grid for each parameter listed in Table 1. These maps broadly demonstrate that most model grid cells are not sensitive to changes in parameter values.

**Table 1:** Parameter Sensitivity Analysis Results, Percent Change in B­[*a*]P Sorbed to Surface Sediments from Pre- to Postflood Simulations Using the 100 Year Flood Baseline Simulation (without Tidal Fluctuation and Future SLR)^a^

				% change (grid)		% change (Eastwick)	
parameter (units)	base value	upper value	lower value	increase (%)	decrease (%)	increase (%)	decrease (%)
total suspended solids boundary concentration (mg L^–1^) at Cobbs and Darby Creeks	50	500	5	–0.78	–0.60	–1.37	–1.26
B[*a*]P boundary concentration (mg L^–1^)	0.0167	0.0835	0.0017	–0.41	–0.65	–0.98	–1.35
partition coefficient (L kg^–1^)	15,000	150,000	1500	–1.73	–0.77	–2.79	–1.45
shear stress multiplier	1	10	0.1	–0.45	–0.68	–1.74	–1.17
upper cohesive shear for deposition (N m^–2^)^b^	0.2	N/A	0.02	N/A	–0.32	N/A	–1.16
exponent for noncohesive resuspension	1	1.5	0.5	–0.63	–0.58	–1.10	–0.92
exponent for cohesive resuspension	1	4	3^f^	–0.39	–0.42^f^	–1.63	–1.47
peak flow^c^ (Darby Creek below OU1^d^, m^3^ s^–1^)	10.82	14.28	5.78	–0.77	–0.08	–2.66	^e^

a The baseline simulation percent change in B­[*a*]P sorbed to surface sediments in the grid was −0.61 and −1.29% in Eastwick.

b For the upper cohesive shear stress for deposition (N m^–2^), the baseline simulation value was already set at the recommended upper limit of the range; therefore, only a sensitivity to decreasing this value is reported.

c Sensitivity analysis simulations are based on the 100 year flood event with fixed downstream tidal elevation, except for the peak flow simulations, where the upper and lower results are based on 500 year and 10 year event simulations, respectively.

d Darby Creek at OU1 (Figure 1) represents the 1D connection at the bridge between the northern and southern portions of the study area.

e The lower scenario for peak flow is based on the 10 year flood event. This event causes flooding and sediment movement on the west side of OU1 but not within the Eastwick neighborhood itself.

f For the exponent for cohesive resuspension, the baseline simulation value was already set at the recommended lower limit of the range; we tested two positive perturbations of this value.

Parameter sensitivity coefficients demonstrate how increases or decreases in parameter values affect changes in the average concentration of B­[*a*]P in surface sediments after the flood event (Table 2). When the sign of the perturbation and the sign of the response match, there is a positive correlation between the parameter and the result. For example, increasing the boundary B­[*a*]P concentration leads to an increase in postevent B­[*a*]P surface sediment concentrations, while the opposite is true for a decrease in this concentration. Sensitivity coefficients are relatively small for the boundary suspended solids and B­[*a*]P concentrations, suggesting low sensitivity, which is important because these are among the more uncertain model inputs. The relatively large sensitivity coefficients for peak flow suggest that the results are more sensitive to the interaction of the initial concentrations of contaminants and the event flows passed from the HEC-RAS model.

**Table 2:** Sensitivity Coefficients (10^3^) and B­[*a*]P Concentrations in Surface Sediments

				Grid		Eastwick	
parameter (units)	base value	upper value	lower value	+	–	+	–
total suspended solids boundary concentration (mg L^–1^)	50	500	5	–0.35	–1.42	–0.09	0.27
B[*a*]P boundary concentration (mg L^–1^)	0.0167	0.0835	0.0017	0.18	–2.01	0.790	–0.69
partition coefficient (L kg^–1^)	15,000	150,000	1500	–1.47	–3.5	–1.69	–2.01
shear stress multiplier	1	10	0.1	0.04	–2.42	–0.51	1.29
upper cohesive shear for deposition (N m^–2^)^a^	0.2	N/A	0.02	N/A	1.78	N/A	1.47
exponent for noncohesive resuspension	1	1.5	0.5	–3.20	–2.16	3.81	7.53
exponent for cohesive resuspension	1	4	3^d^	1.06	0.82^d^	–5.03	–2.75^d^
peak flow^b^ (Darby Creek below OU1^c^, m^3^ s^–1^)	10.82	14.28	5.78	–9.87	9.09	–43.6	28.0

a For the upper cohesive shear stress for deposition (N m^–2^), the baseline simulation value was already set at the recommended upper limit of the range; therefore, only a sensitivity to decreasing this value is reported.

b Sensitivity analysis simulations are based on the 100 year flood event with fixed downstream tidal elevation, except for the peak flow simulations, where the upper and lower results are based on 500 year and 10 year event simulations, respectively.

c Darby Creek at OU1 (Figure 1) represents the 1D connection at the bridge between the northern and southern portions of the study area.

d For the exponent for cohesive resuspension, the baseline simulation value was already set at the recommended lower limit of the range; we tested two positive perturbations of this value.

### 3.3 Sediment Transport

Sediment scour and deposition involve both net scour (or deposition), where the sediment volume changes in a grid cell, and short-term exchanges, where sediment is temporarily resuspended, equilibrates with contaminants already in suspension, and is redeposited within the same model grid cell. The latter process could result in changes in the sediment contaminant concentration without major changes in the sediment volume. At the LDCA scale, the general patterns of sediment scour and deposition in the compound flood event with 100 year flood, MHHW tide, and SLR simulations result in a net accumulation of sediment in the LDCA system. Net sediment deposition is +40,891 m^3^ without SLR and decreases with future SLR to +40,487 m^3^ (RSL +0.31 m) and +37,911 m^3^ (RSL +0.68 m).

The capped portions of OU1 and OU2 landfills were not inundated in any of the simulations and, therefore, were not subject to sediment scour and deposition (Figure 4). The boundaries of the OUs extend beyond the edges of the landfill caps, e.g., the boundary for OU1 extends to the thalweg of Darby and Cobbs Creeks, and include areas where the OUs are flooded even if the capped landfill sections are not. Sediment deposition occurs primarily in the lower-gradient portions of Lower Darby Creek in and near Tinicum Marsh. This sediment is derived largely from scouring of the channel bed and banks near Clearview Landfill (OU1) and from tributary sediment inputs. The SLR and the increase in the Delaware River water surface elevation boundary increase the extent, depth, and duration of inundation while reducing the energy grade in Tinicum Marsh and the lower segments of Darby Creek, resulting in a slight decrease in the total sediment retention in the system. SLR has little effect on changes in sediment dynamics in the OU1 area, as the channel water surface elevation in these areas will have only minor impacts from tidal boundaries because the bottom elevation is near the normal tidal range for the downstream boundary.

**Figure 4:**
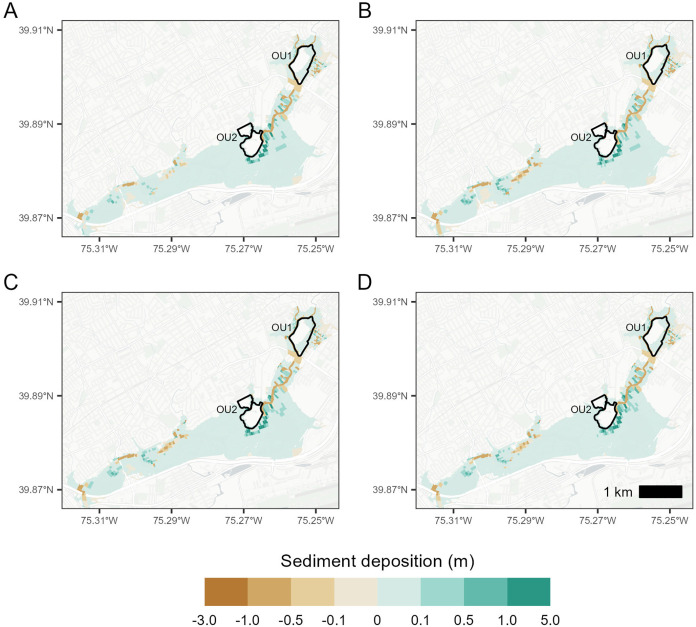
Change in surface sediment depth per cell area (m^3^ m^–2^), pre- vs postevent simulation for (a) 100 year flood, (b) 100 year flood with MHHW, (c) 100 year flood with MHHW and +0.31 m future RSL, and (d) 100 year flood with MHHW +0.68 future RSL.

### 3.4 Benzo­[*a*]­Pyrene Transport

Individual segments within the model domain undergo a complex set of changes in surface water conditions (volume, velocity, shear stress), sediment volume, and contaminant concentrations throughout the flood simulation. For example, time series results at the Eastwick model cell (location identified in Figure 1) for the water column and underlying surface sediment are shown in Figure 5 during the simulated 100 year flood. This model grid cell is located among the buildings of the Eastwick neighborhood, east of OU1, and is prone to inundation during the 100 year flood. Velocity and shear stress increases in this grid cell affect the sediment from the start of the flood at hour 16 of the simulation, peaking at hour 24. The shear stress is not high enough to cause sediment erosion within the grid cell because it does not exceed the 2 N m^–2^ threshold for erosion to occur; however, when shear stress is greater than 0.2 N m^–2^, sediment deposition is limited, and particles remain suspended. This results in the overall deposition of sediment in the grid cell.

**Figure 5:**
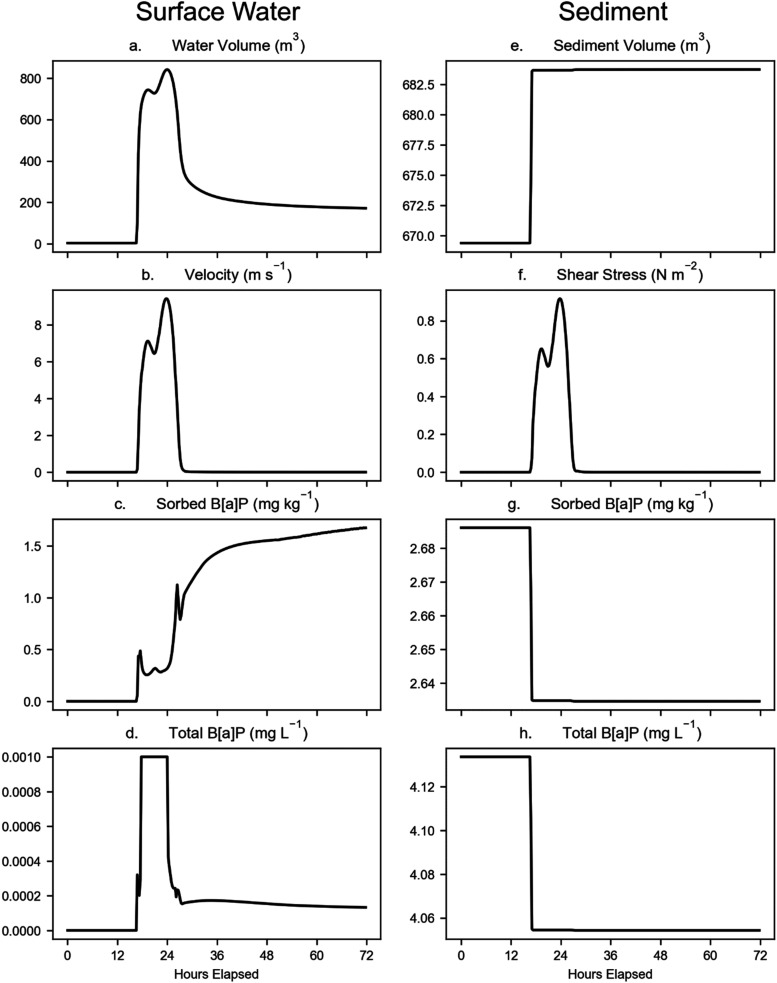
Flood, sediment, and B­[*a*]­P WASP results (100 year flood with MHHW) for the Eastwick model cell (see Figure 1) for the water column and underlying surface sediment. Note the differing units and *y*-axes for each panel.

Suspended solid concentrations in water increase at the start of the 100 year flood event and are dominated by resuspended sands and then decline (Figure 6). Sediment deposition occurs primarily at the start of the event before shear stress exceeds the deposition threshold, with a small amount of additional deposition occurring at the end of the event, while the total B­[*a*]P concentration in the water column is the highest during the rising limb of the event (Figure 5). The total B­[*a*]P concentration in water is greatest near the start of the event due to load from upstream, then declines; however, the B­[*a*]P concentration in suspended sediment remains high as suspended sand and silt settle, leaving smaller clay particles with attached B­[*a*]P in the water column. While additional B­[*a*]P is deposited with the new sediment, the B­[*a*]P concentration in this depositional material is less than that B­[*a*]P concentration in the benthic sediment, so the net effect is a dilution of concentration in the sediment as the sediment volume increases.

**Figure 6:**
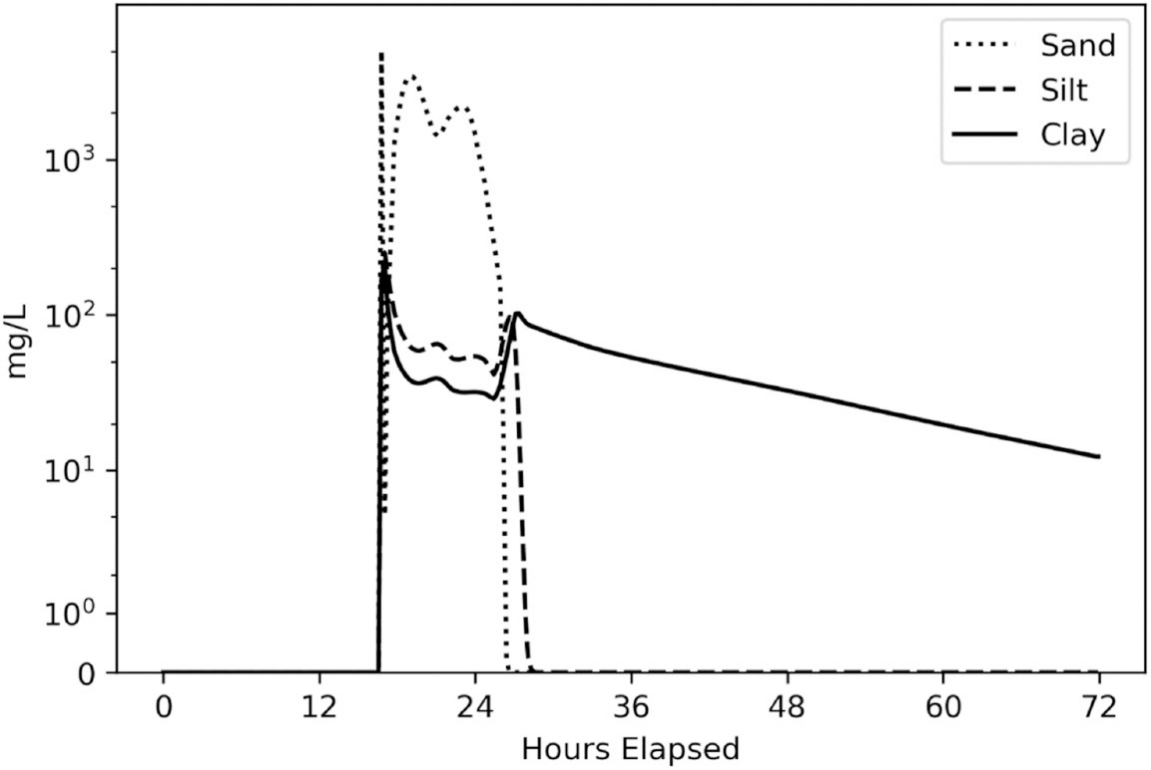
Suspended solids concentration in surface water for WASP results (100 year flood with MHHW) for the Eastwick Model Cell (see Figure 1).

Sensitivity of B­[*a*]P concentrations to SLR is summarized in terms of the change in B­[*a*]P concentrations in surface sediment over the course of the 100 year flood (Table 3). Because B­[*a*]P is highly particle reactive, the sensitivity of concentration results and their spatial distribution depend on sediment scour and deposition processes (Figure 6). Average B­[*a*]P concentrations decline in all scenarios due to dilution by sediment with lower B­[*a*]P concentration during flooding, coupled with SLR, slowing velocities in the lower portion of Darby Creek and increasing overall deposition of this sediment. However, the difference in concentration caused by SLR is minimal in the Eastwick area due to the higher base elevations of the thalweg in this portion of Darby Creek; the SLR scenarios tested here have little impact on this part of the LDCA.

**Table 3:** Simulated Changes in B­[*a*]P in Surface Sediment (%) Postflooding Following the Combined Fluvial and SLR Scenarios

scenario	% change (model domain)	% change (Eastwick)
100 years, MHHW	–0.67	–1.22
100 years, MHHW, RSL +0.31 m	–0.69	–1.22
100 years, MHHW, RSL +0.68 m	–0.85	–1.23

The net result of the simulated contaminant transport across the LDCA reflects a balance between the redistribution of contaminants within the initial grid and the influx of contaminants from the tributaries (Figure 7). The differences in B­[*a*]P redistribution patterns are minor across the SLR scenarios, which is consistent with the differences in the sediment redistribution patterns (Figure 4). Ultimately, SLR has a limited effect on the 100 year flood-induced transport of sediments and associated B­[*a*]P in the LCDA; the 100 year fluvial flood routed from the Darby Creek watershed is the dominant driver of sediment and B­[*a*]P transport in the system. The greatest increases in B­[*a*]P concentration are in the east and southeast of Tinicum Marsh. This is the result of large volumes of sediment containing high B­[*a*]P concentrations south of OU1 (Figure 2) being transported and then deposited within Tinicum Marsh, which initially has low B­[*a*]P concentrations.

**Figure 7:**
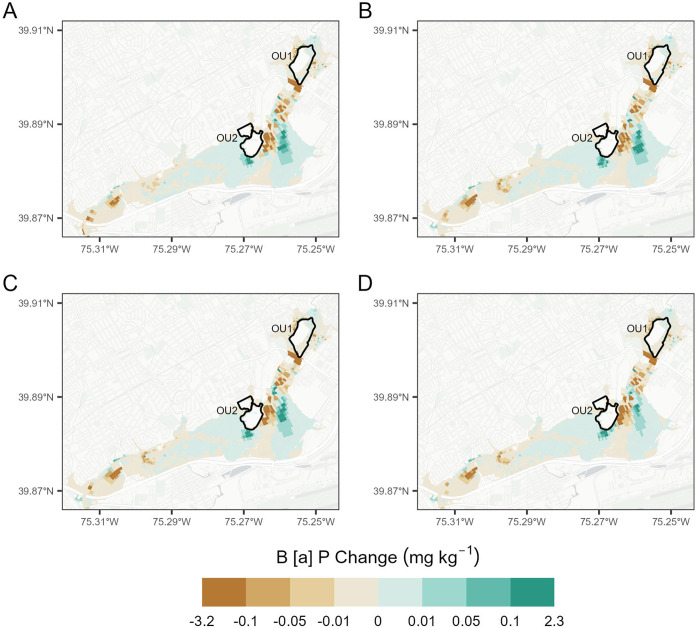
Change in B­[*a*]P in surface sediment (mg kg^–1^), pre- vs postevent simulation for (a) 100 year flood, (b) 100 year flood with MHHW, (c) 100 year flood with MHHW and +0.31 m RSL, and (d) 100 year flood with MHHW +0.68 RSL.

### 3.5 Implications for LDCA and Other Contaminated Sites

Previous studies have used PAH sediment sampling coupled with flood modeling to infer locations of potential contaminant deposition in floodplains.^15^ This research takes the next step in employing a coupled mechanistic modeling approach to better understand the transport of PAH during extreme flooding. We also demonstrated that we can successfully model compound flooding and develop projections of contaminant transport under future conditions. Based on our results, flooding and sediment distribution patterns are complex in the LDCA system and do not mirror B­[*a*]P mobilization and redistribution. B­[*a*]P concentration patterns prior to flood onset and after the flood event differ as a function of initial B­[*a*]P concentrations in sediment and tributary inputs of B­[*a*]­P. As relatively low concentration B­[*a*]­P-sorbed sediment is deposited in areas of high B­[*a*]P concentrations, the concentrations in benthic sediment decline and vice versa. Hence, flood modeling/mapping and/or sediment transport alone are not reliable proxies for flood-induced B­[*a*]P transport patterns.

The simulations demonstrated that sediment and contaminant transport in the LDCA and surrounding river floodplains are largely driven by fluvial flooding; SLR and MHHW are minor compounding drivers of transport dynamics. This is not unexpected given the contrast in stage changes due to fluvial flooding and SLR, with a +5 m increased stage in a short period of time at the confluence of Darby and Cobbs Creeks due to the fluvial flood compared to the +0.68 m chronic increase at the Delaware River. The site characteristics, particularly Tinicum Marsh, are also important. The marsh acts as both a flood barrier and a sediment and contaminant sink. As a barrier, it absorbs most of the chronic inundation from tidal cycles and SLR from the Delaware River and Delaware Bay, shielding upstream LDCA from these effects. Conversely, Tinicum Marsh slows Darby Creek floodwaters and promotes sediment deposition that likely contains B­[*a*]P transported upstream, impacting the aquatic ecosystem within Tinicum Marsh and John Heinz National Wildlife Refuge. Many contaminated sites are situated among estuarine and coastal wetland systems such as these; this demonstrates both their importance in both protection from coastal and fluvial flood events and potential to act as a contaminant sink with negative implications for the aquatic ecosystem.

The simulation results presented here were submitted to the Environmental Protection Agency National Remedy Review Board to inform their advisement on feasibility planning of LDCA Aquatic System OU4, established in 2021. The OU4 has a larger spatial extent, encompassing aquatic life within the creeks, Tinicum Marsh, and John Heinz National Wildlife Refuge. Indeed, a proposed future sampling campaign will include pre- and poststorm event soil and sediment B­[*a*]P sampling and storm event-based sampling of suspended sediment and sorbed B­[*a*]P in key areas of erosion and deposition (Figures 4 and 7). This will refine the models produced here and guide the next stage of remediation and operation of LDCA while considering future compound flood events.

These models, particularly HEC-RAS (for regulatory and nonregulatory products), have been widely implemented across much of the United States. The Federal Emergency Management Agency’s Future of Flood Risk Data Initiative is aiming to produce models of every four-digit hydrologic unit code watershed, for which HEC-RAS is the standard operating hydraulic model.^61^ We envision broader efforts to couple that effort with WASP for modeling future compound flooding events at the thousands of contaminated sites in the country to better quantify and mitigate site- and community-level impacts of these events. However, there will still need to be site-based considerations on how to validate these models; environmental sampling at these sites is in support of remediation activities and not necessarily model evaluation, as was the case with LDCA.

### 3.6 Limitations

Extensive temporal and spatial data of initial conditions and boundary conditions are critical in the WASP model setup and interpretation of results. Available measurement data relevant to this study at LDCA span two decades across multiple sampling campaigns that have quantified soil and sediment physical and chemical characteristics in support of past and ongoing remediation activities. Major flood events (e.g., tropical storm Isaias in 2020) that have occurred since the latest round of sampling may have shifted the areas of high and low concentrations of B­[*a*]­P within the system. Surface water sampling data at LDCA are limited in time, with few repeated measurements and none during storm events. Further, there are five upstream inputs (streams) to the study area, all of which drain highly urbanized watersheds with a history of industrial activity but with similarly limited data.

The paucity of time series water quality data in Darby Creek, especially during storm events, hampers our ability to validate in-stream sediment and B­[*a*]P transport. However, WASP has been used for decades to simulate a broad suite of particle reactive chemical solutes..^30,31,33,35,36^ While the lack of data presents uncertainty in the results, the sensitivity tests and scenario analysis of the coupled HEC-RAS/WASP mechanistic modeling framework highlight how the system responds to external changes and will guide further sampling and remediation planning, as previously discussed.

## 4 Conclusions

The one-way coupling of HEC-RAS and WASP to simulate compound flooding (fluvial, tidal, and SLR) and B­[*a*]P transport demonstrated that changes to the LDCA system in these compound events will continue to be dominated by extreme fluvial flooding, with the capability to erode B­[*a*]­P-laden sediment. Over time, these processes may deplete the B­[*a*]P in the upstream part of the system, leading to accumulation in Tinicum Marsh. However, SLR coupled with tidal cycles can alter flooding and contaminant transport dynamics in the lower reaches of Darby Creek, from the Delaware River to downstream of OU1. Deposition of lower concentrations of B­[*a*]P in suspended sediment from upstream, although greater than area background concentrations, may result in decreases in B­[*a*]P concentrations in benthic sediment.

The modeling framework, scenarios, and simulations we developed can be used as additional data to inform the remediation and planning process, especially as remedial site managers plan for future conditions. A significant number of Superfund sites and other contaminated and hazardous waste sites in the United States are potentially prone to single or compounded fluvial flood, storm surge, or sea-level and tidal-driven nuisance inundation events.^5,6^ These compound events are often overlooked and understudied.^17,19^ Our findings illustrate the need and provide an approach to conduct site-specific model-based assessments of compound flood impacts on contaminant fate and transport. This is critical for remediation, planning, and adaptation to future conditions at the contaminated sites.

## Supplementary Material



## Notes

Additional details on HEC-RAS and the WASP model setup and parametrization and spatial results of the WASP sensitivity analysis (PDF)
