# Supplementary material for: Compound Impacts of Fluvial Flooding and Sea-Level Rise on Benzo[a]pyrene Transport in the Lower Darby Creek Area Superfund Site, Pennsylvania, USA
Source: ACS ES T Water. 2025 Jun 20;5(7):3613–27. doi: 10.1021/acsestwater.4c00814 (PMC12261278; doi:10.1021/acsestwater.4c00814)
Supplement: Supplementary file 1 [file ew4c00814_si_001.pdf]

Title: Compound impacts of fluvial flooding and sea-level rise on benzo[a]pyrene transport at the Lower Darby Creek Area, Pennsylvania, USA Superfund Site

SUPPORTING INFORMATION

Sean A. Woznicki[1], Josh Barber[2], Jonathan Butcher[3], Jonathan Essoka[4], Maureen Harris[5], Megan Mehaffey[6], Bruce Pluta[2], Afshin Shabani[3], and Pai-Yei Whung[7]

[1]Annis Water Resources Institute, Grand Valley State University, 740. W. Shoreline Dr., Muskegon, MI, USA, 49441, Email: woznicse@gvsu.edu

[2]Superfund Emergency Management Cleanup Division, Region 3, U.S. Environmental Protection Agency, Email: barber.josh@epa.gov; pluta.bruce@epa.gov

[3]Tetra Tech, Inc., Email: jon.butcher@tetratech.com, afshin.shabani@tetratech.com

[4]Center for Environmental Solutions and Emergency Response, Office of Research and Development, U.S. Environmental Protection Agency, Email: essoka.jonathan@epa.gov

[5]AECOM, Email: maureen.harris@aecom.com

[6]Center for Public Health and Environmental Assessment, Office of Research and Development, U.S. Environmental Protection Agency, mehaffey.megan@epa.gov

[7]Center for Environmental Measurements and Modeling, Office of Research and Development, U.S. Environmental Protection Agency (retired, paiyei.whung@gmail.com)

1 HEC-RAS SET-UP

1.1 Data

We used HEC-RAS 2D unsteady flow model to simulate flood inundation of 100-year flood event and to produce time series of flow depth, flow velocity, and shear stress in the LDCA. Initial data sources were obtained from reports for the study sites to support hydraulic modeling (Golder Associates, Inc., 2018, Tetra Tech, 2018) and 1-meter resolution of aerial orthophotos, spatial data identifying the geographic extent of OU1 and OU2, City of Philadelphia 2015 bare earth LiDAR and associated digital elevation model (DEM; 1-meter resolution) for the area of study. Elevation data form the basis for the terrain model, which allowed configuration of the 2D flow areas including the 2D computational mesh and bathymetric survey data collected to support the OU2 Remedial Investigation/Feasibility Study (EA Engineering, 2012)

1.2 Model Grid Configuration

The configuration of 2D flow encompasses area from the upstream end of OU1 to the bridge crossing downstream of OU2 (Wanamaker Avenue) to simulate 2D flood inundation and hydraulics (including water depth, velocity, and shear stress) across the principal area of interest. However, as HEC-RAS 5.07 is limited for computations of bridge hydraulics in 2D areas, our model domain is also composed 1D modeling for bridges whose decks obstruct flow during 100-year in order to achieve the full bridge modeling capabilities in HEC-RAS. We developed three discrete 2D flow areas, one upstream and one downstream of the bridge at Hook Road/84th Street and connect them with a 1D river reach, a third downstream-most 2D flow area extends from the Wanamaker Avenue bridge to just upstream of the railroad bridge, where the model transitions back to 1D. This will allow for modeling of bridge hydraulics within the 1D reaches of the overall model domain.

1.3 Tidal and Sea Level Rise (SLR) Boundary Conditions

NOAA tide predictions comprise the principal data source for conducting the tidal analyses, i.e., Philadelphia and Billingsport tide stations. (https://www.tidesandcurrents.noaa.gov/map/index.html?region=Pennsylvania#). NOAA references Billingsport tides to the Philadelphia station based on the following relationships: *Billingsport (8538552) → Philadelphia (8545240)*, *Time offsets (minutes; additive) → high: -35 low: -28* and *Height offsets (feet; multiplicative) → high: 0.93 low: 0.95*. We applied the Billingsport offset to the tide record for Philadelphia to develop representative tidal boundary conditions at the mouth of Darby Creek.

For the sea-level rise (SLR) analysis, we used the 50th percentile Philadelphia tide station global mean sea level height increase-scenario results of 0.5 m and 1.5 m to project 2050 SLR (Sweet et al. 2017). The sea level rise scenarios evaluated in this study utilized the mean high water (MHHW) NOAA tidal elevation for Philadelphia (Station 8545240) of 3.19 ft, relative to NAVD88. The MHHW peak is aligned to occur at 24 hours into the simulation and the total simulation length is 72 hours. The static sea level rise increase is imposed to construct each SLR scenario (i.e., 0, 0.5 m and 1.5 m).

The baseline for the storm surge scenarios the MHHW datum for the downstream tidal boundary. According to NOAA, MHHW represents:

> *"…The average of all the high water heights observed over the National Tidal Datum Epoch. For stations with shorter series, comparison of simultaneous observations with a control tide station is made in order to derive the equivalent datum of the National Tidal Datum Epoch."*

In other words, MHHW is calculated from observed tidal time series during the tidal epoch. There is no unique reporting date for MHHW; in fact, it is likely to occur dozens of times over the course of a tidal epoch. An epoch is a 19-year tidal cycle used to calculate datums; the current official epoch (National Tidal Datum Epoch, or NTDE) spans 1983 through 2001. However, the Philadelphia station to which the tide offsets to Billingsport are calculated (8545240) did not start collecting data until 1989. Therefore, the MHHW datum reported on the Philadelphia station home page was estimated by NOAA using the Reedy Point, DE station as the "control tide station" to supplement development of the complete NTDE time series needed to estimate the datums.

Due to the unavailability of tide records at Philadelphia gage 8545240 prior to 1989, we based our analysis on the 1990-2008 period, which represents the closest available complete 19-year record to the current official NTDE (1983-2001). Initially, Tetra Tech considered reviewing the high and low tide records spanning 1990-2008 to identify magnitudes matching MHHW to construct tidal time series surrounding MHHW. However, upon further analysis, we discovered that there was considerable variation among time series leading up to and following the multiple occurrences of MHHW between 1990-2008. To address this, we identified all high tide values during the 1990-2008 time period equal to the reported MHHW datum. We then retrieved the high and low tide magnitudes and date/time values spanning 1.5 days prior to and 3.0 days following the identified MHHW and calculated the median magnitudes and time offsets among this population of observations. The median magnitudes and time offsets were calculated individually for three high/low cycles preceding and six high/low cycles following the MHHW matches. The Philadelphia-to-Billingsport translation was then applied to the median MHHW high/low time series, and a sine wave function was used to calculate the intervening values between the high and low tides in 5-minute increments to construct the dynamic tidal boundary condition for the mouth of Darby Creek for HEC-RAS. Given the location of points A (x_1,y_1) and B (x_2,y_2) on a sine curve where the amplitude is y_2-y_1 and x_1 and x_2 are points in time the equation of a half-sine curve that passes through both points A and B is in Equation 1.

$$f(x) = \frac{y_2-y_1}{2}\cos\frac{\pi(x-x_2)}{|x_2-x_1|} + \frac{y_1+y_2}{2} \quad (1)$$

2 HEC-RAS TO WASP LINKAGE

2.1 External Boundary Flows (HEC-RAS QNet Output)

HEC-RAS 2D produces an undocumented "QNet" output series in the BMD2 file. This holds the external boundary flows, which are primarily associated with virtual model segments external to

the model grid that are then connected to segments within the model domain and represent inflows to and outflows from that domain as flows across the face joining the internal and virtual external segment. HEC-RAS also redirected parts of the external boundary flows to nearby cells within the grid, likely to enhance model stability. These flows need to be restored to the external boundary flows specified in WASP to achieve flow mass balance in the coarser WASP grid cells. Figure S1 shows an example in which part of the flow entering the OU1 grid is represented as a direct inflow to interior cells 5,818 instead of being represented as a link from an external virtual cell.

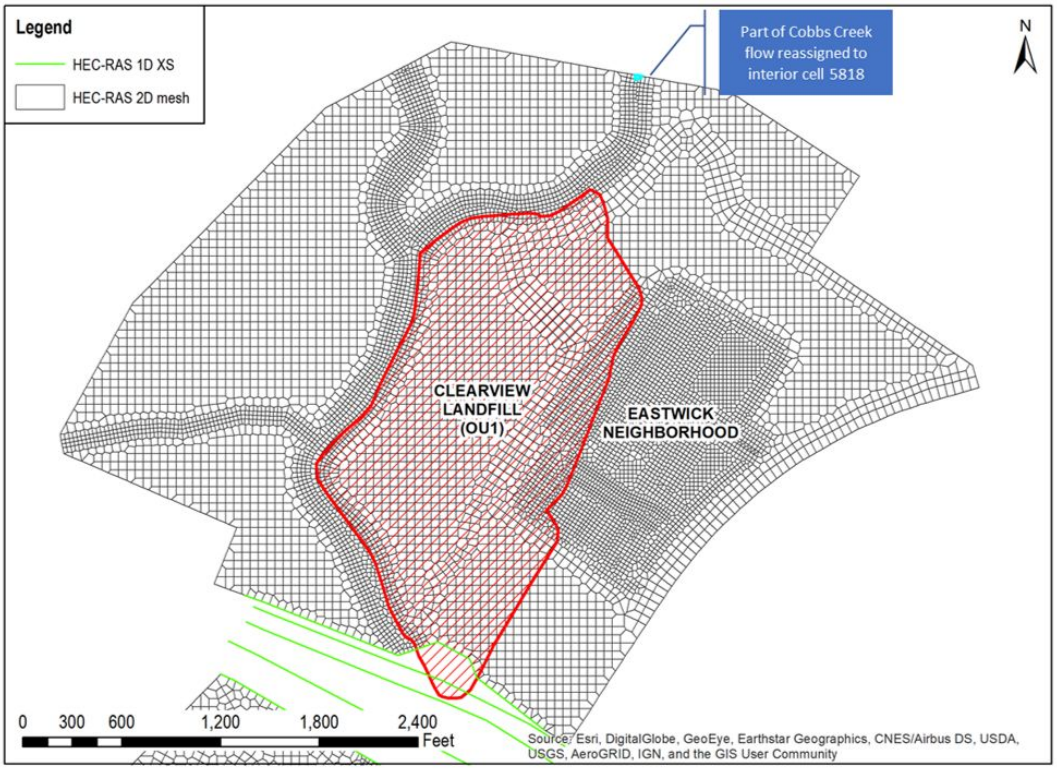

Figure S1. Example of QNet redirection of external flows from Cobbs Creek to HEC-RAS internal grid cell 5,818.

HEC-RAS assigns a value of QNet to every internal grid cell at every time step; however, the majority of these flows are zero. To avoid unnecessary extra processing, we address this by including only those QNet flows where the total inflow sums to 25 m^3 or more over the course of the 72-hour flood event simulation (equivalent to an average flow of 0.000385 cfs) in the WASP hydrodynamic file.

2.2 Linkage Process

Data for WASP is extracted from the HEC-RAS model output (with computation-level output turned on) without modifying any of the HEC-RAS output files. Two types of information must be transferred from HEC-RAS to WASP: (1) static information regarding the grid, grid-cell geometric properties, and the relationships between the HEC-RAS and WASP grids, and (2) unsteady timeseries information on flows and volumes. The HDF file uses a parsimonious space-saving format in which the cell or face number is given by the row index of a vector, while

various properties (such as the relationship between water surface elevation and volume) are stored in continuous vectors where the cell or face position within the vector is indexed in a separate table that specifies entry point and count. Volume is a property of a grid cell, while flow is a property of the multiple faces of each grid cell, so the indexing between cells and faces is also crucial.

The following static data are retrieved for the 2D areas

- From HD5 path **Geometry – 2D Flow Areas – [area name]**:
  - Cells Surface Area
  - Cells Volume Elevation Info (pointer index to next table)
  - Cells Volume Elevation Values
  - Faces Cell Indexes (relationship between faces and cells)
  - Boundary Condition Lines (location assignments of internal boundary conditions)

The timeseries output data for the 2D areas are:

- From HD5 path **Results – Unsteady – Output – Output Blocks – Base Output - Unsteady Time Series – 2D Flow Areas – [area name]:**
  - Face Velocity
  - Face Flow (calculated by HEC-RAS 2D postprocessor option)
  - Water Surface Elevation (above cell base)
  - Q Net: Internal flow adjustments made by HEC-RAS
- From HD5 path **Results - Unsteady - Output - Output Blocks - Base Output - Unsteady Time Series - 2D Flow Areas – [area name] - Boundary Conditions**
  - Flow (for each boundary condition line specified as “internal” in the geometry.

For the 1D areas, output is arranged by cross section. There is again static geometry data:

- From HD5 path **Geometry – Cross Sections – Flow Distribution**, retrieve
  - Info (lookup pointer for water surface
  - Water Surface Elevation (minimum elevation of the cross section, NAVD88)
- From **DSS file storage**, retrieve
  - Storage-Flow table for each cross-section

Timeseries outputs retrieved for the 1D cross-sections are:

- From HD5 path **Results – Unsteady – Output – Output Blocks – Base Output – Unsteady Time Series - Cross Sections**:
  - Flow
  - Velocity Total
  - Water Surface (stage, NAVD88)

The transfer from HEC-RAS to WASP is complex and is handled using a modular approach to allow intermediate checks and debugging. The code for these tools is presented in the appendix. The tools are run in sequential order from the command line or batch file:

1. Get_hdf_data.py: Python code to extract selected data from the HEC-RAS HD5 file and write it to intermediate text files.

2. HD5process.f90: FORTRAN 90 code that takes the extracted HD5 data for the 2D areas and converts it to the WASP grid and proper units and format for constructing the WASP hyd file.
3. HD51-D.f90: FORTRAN 90 code to process HD5 and DSS data for the 1D areas. (Note that it is necessary to provide a limited amount of geometric information from the DSS file for this process, which has not been automated at this point.)
4. Hydrolink_v23_sed.f90: FORTRAN 90 code that uses the hydrolink API to process the output of the previous steps and convert it to a WASP hyd file. This code also sets up a representation of sediment cells underlying each water column cell and passes sediment volume information to the hyd file. Note that the current version of WASP does not correctly specify these cells as "surface benthic" but instead generates them as "subsurface water." This designation must be changed by hand in the WASP interface.

Steps 2 through 4 each have a user-specified control file (with file name passed as an argument) that controls input and output file names and other information. The control files also contain links to a series of additional files that describe the relationship between HEC-RAS and WASP cells and faces. These were developed in ArcGIS. HEC-RAS cells are assigned to WASP cells based on the location of the HEC-RAS cell centroid relative to the WASP index grid. HEC-RAS face flows that correspond to boundaries between WASP cells are identified by filtering the full set of HEC-RAS faces to retain those that join HEC-RAS cells that belong to different WASP cells. Steps 2 and 3 are each executed twice in order to Hydrolink_v3_sed to construct a single WASP hyd file.

To transfer QNet flows properly to the WASP model, each internal QNet flow is reassigned to the corresponding channel or link connecting the receiving HEC-RAS cell to a virtual external cell. These links are specified in the control file by HEC-RAS cell number and face number so that the FORTRAN program can tabulate their contribution to fluxes across WASP cell boundaries along with the other flows represented by HEC-RAS cell linkages.

3 WASP SET-UP

3.1 Model Grid and Wet/Dry Cells

The WASP grid is aggregated to a larger spatial scale and a longer model time step than the HEC-RAS grid due to computational time constraints. For OU1, we used the HEC-RAS interface to generate a coarser model grid, then grouped the HEC-RAS grid cells whose centroid fell within each coarser WASP grid cell. The two HEC-RAS autogenerated grids were less aligned in OU2 area. For OU2, we generated a curvilinear-orthogonal index grid at the desired coarser spatial resolution and used that to assign HEC-RAS cells to the WASP grid. The curvilinear-orthogonal grid was generated using the Visual EFDC software package. WASP grid contains cells that wet and dry (or are always dry) during certain events. However, WASP will crash when cell volume goes to zero and WASP water quality simulation will also slow drastically as volume approaches zero. In order to address this issue, the code for creating the WASP hydrodynamic linkage (".hyd") file specifies when a WASP cell is simulated as almost dry. The cell will then be set to (1) a minimum volume of 4 m^3, (2) a depth of greater that volume divided by area and 0.01, and (3) velocity within the segment of 0.

3.2 WASP Parameterization

3.2.1 Sediment parameterization

The WASP model specifies sediment concentrations on a volumetric basis. For the dynamic sediment bed option used with process-based sediment simulation, WASP preserves the initial bulk density and porosity of sediment segments while changing the volume in response to fluxes of different size classes. Porosity (φ) is equal to $1 - P_b/P_d$, where P_b is the bulk density and P_d is the particle density. We assume bulk densities of 1.905, 1.833, and 1.76 g/mL for sand, silt, and clay, respectively, and particle densities of 2.65, 2.55. and 1.27 g/mL (Engineering Toolbox, 2010). As $Pb = (1 - \rho) \times Pd$, the volumetric concentration of sediment class *i* is then $f_i \times Pb_i$ (converted to mg/L for WASP) and the calculation can be verified by reproducing the observed porosity.

Initial values for parameters describing the transport, scour, and deposition of sediment were set based on WASP guidance and examples. Most notably, these include the default values and ranges contained in Ambrose et al. (2017) and Ambrose and Wool (2017); presentation material and examples from the WASP Advanced Toxicants Training courses held in 2017 and 2018, and a detailed example application of WASP8 to mercury transport in the Sudbury River, Massachusetts. Solids transport constants are presented in Table S1.

Table S1. Solids Transport Constant Parameters for Lower Darby Creek Baseline Application

Parameter	Sand	Silt	Clay
Diameter (mm)	1.0	0.033	0.004
Critical Cohesive Sediment Fraction	0.2	0.2	0.2
Critical Shear for Erosion of Cohesive Bed (N/m^2)	2.0	2.0	2.0
Shear Multiplier for Cohesive Resuspension	5.0	1.0	1.0
Shear Exponent for Cohesive Resuspension	3.0	1.0	1.0
Critical Shear for Non-cohesive Resuspension	1.0	1.0	1.0
Shear Exponent for Non-cohesive Resuspension	1.5	1.5	1.5
Shear Multiplier for Non-cohesive Resuspension	1.0	1.0	1.0
Bed Load Multiplier	1.0	1.0	1.0
Lower Critical Shear Stress for Solid Deposition (N/m^2)	0	0	0
Upper Critical Shear Stress for Solid Deposition (N/m^2)	0.2	0.2	0.2
Shear Exponent for Deposition	1.0	1.0	1.0

3.2.2 Soil and Sediment Sample Locations and Decision Units

Surface sediment contaminant concentrations were collected across multiple sampling campaigns between 2002 and 2016 (Tetra Tech NUS, 2011; EA, 2016; Golder Associates, 2018). The results from these campaigns consist of individual samples and Composite Decision

Units (DUs) (Figure S2) and were integrated to establish initial conditions of B[a]P in the WASP model. Each of the 35 DU is representative of 50 randomly sampled locations from a regular grid within the DU and composited for analysis (EA, 2016).

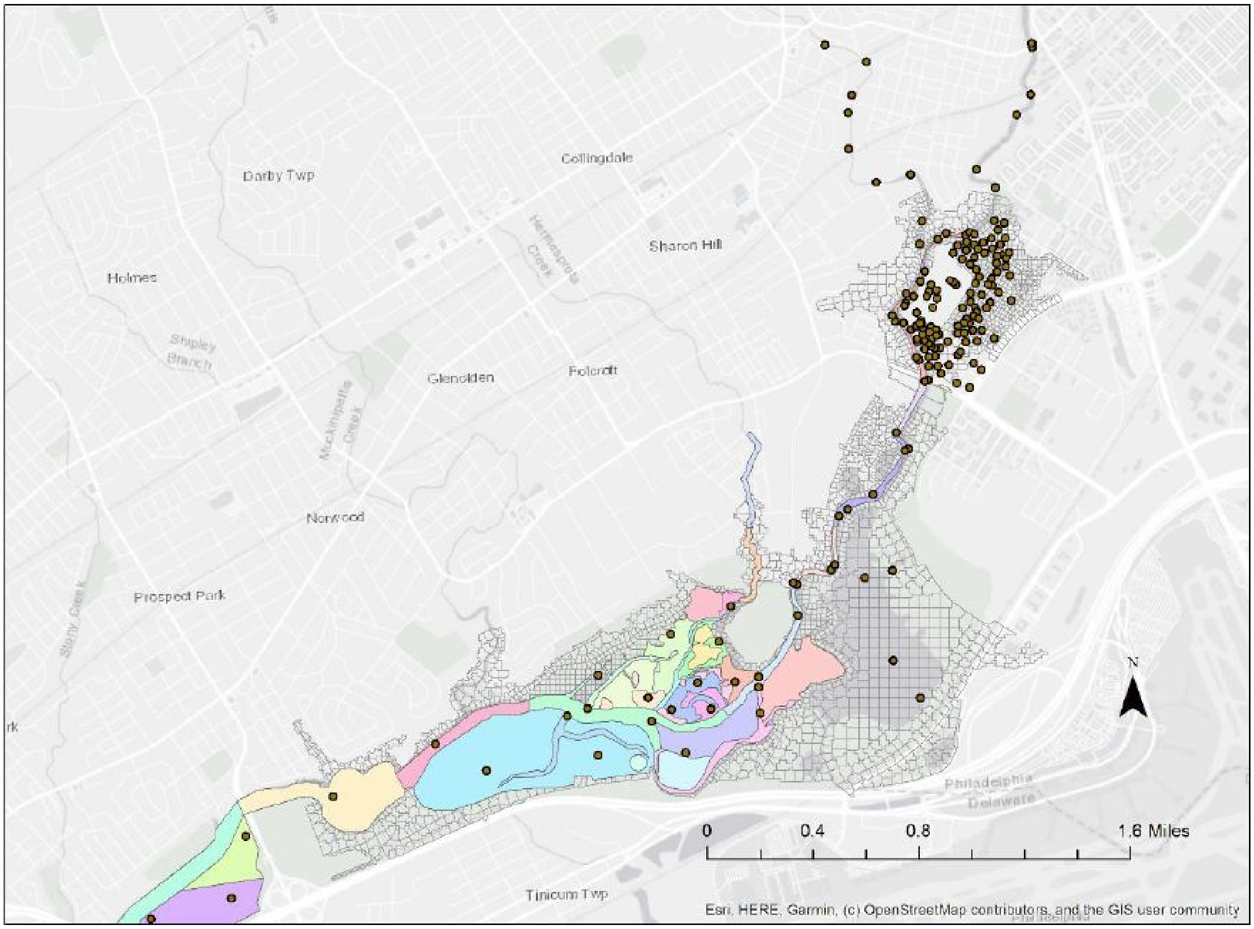

Figure S2. Sediment sampling locations (points) and Composite Decision Units (colored polygons).

4 MODELING RESULTS

4.1 HEC-RAS Results

Table S2 and Table S3 highlight the differences in flood depth across the HEC-RAS model grid between scenarios. Table 2 compares the 100-year flood with static water level condition (no tidal cycle) to the remaining scenarios. Table 3 compares the MHHW tide + 100-year flood to the sea level rise scenarios.

Table S2. Summary of flood depth differences (m) from the 100-yr fluvial flood, downstream static tide level scenario across the model grid

Scenario	Max. decrease (m)	Max. increase (m)	Mean change (m)	Standard dev. (m)
100-yr, MHHW	-0.98	0.55	-0.04	0.06
100-yr, RSL+0.31 m	-0.43	0.54	0.04	0.09
100-yr, RSL+0.68 m	-0.34	1.22	0.2	0.21

Table S3. Summary of flood depth differences (m) from the 100-yr fluvial flood, MHHW scenario across the model grid

Scenario	Max. decrease (m)	Max. increase (m)	Mean change (m)	Standard dev. (m)
100-yr, RSL+0.31 m	-0.34	0.75	0.07	0.1
100-yr, RSL+0.68 m	-0.34	1.66	0.23	0.23

4.2 WASP Sensitivity Analysis

Figures Figure S3 through Figure S10 show the spatial results of the sensitivity analysis for each parameter listed in Table X of the main text. For each adjusted parameter, the percent difference in end of simulation B[a]P in sediment concentration with respect to the base parameter set simulation's ending B[a]P concentration in sediment is mapped, for each WASP model grid cell (Equation S1, where *i* is the parameter and *j* is the grid cell). Note that the static tidal boundary condition + 100-year flood simulation was used to perform the sensitivity analysis.

$$Percent\ Difference_{i,j} = \frac{B[a]P_{i,j,end} - B[a]P_{base,j,end}}{B[a]P_{base,j,end}} \times 100 \qquad \text{(S1)}$$

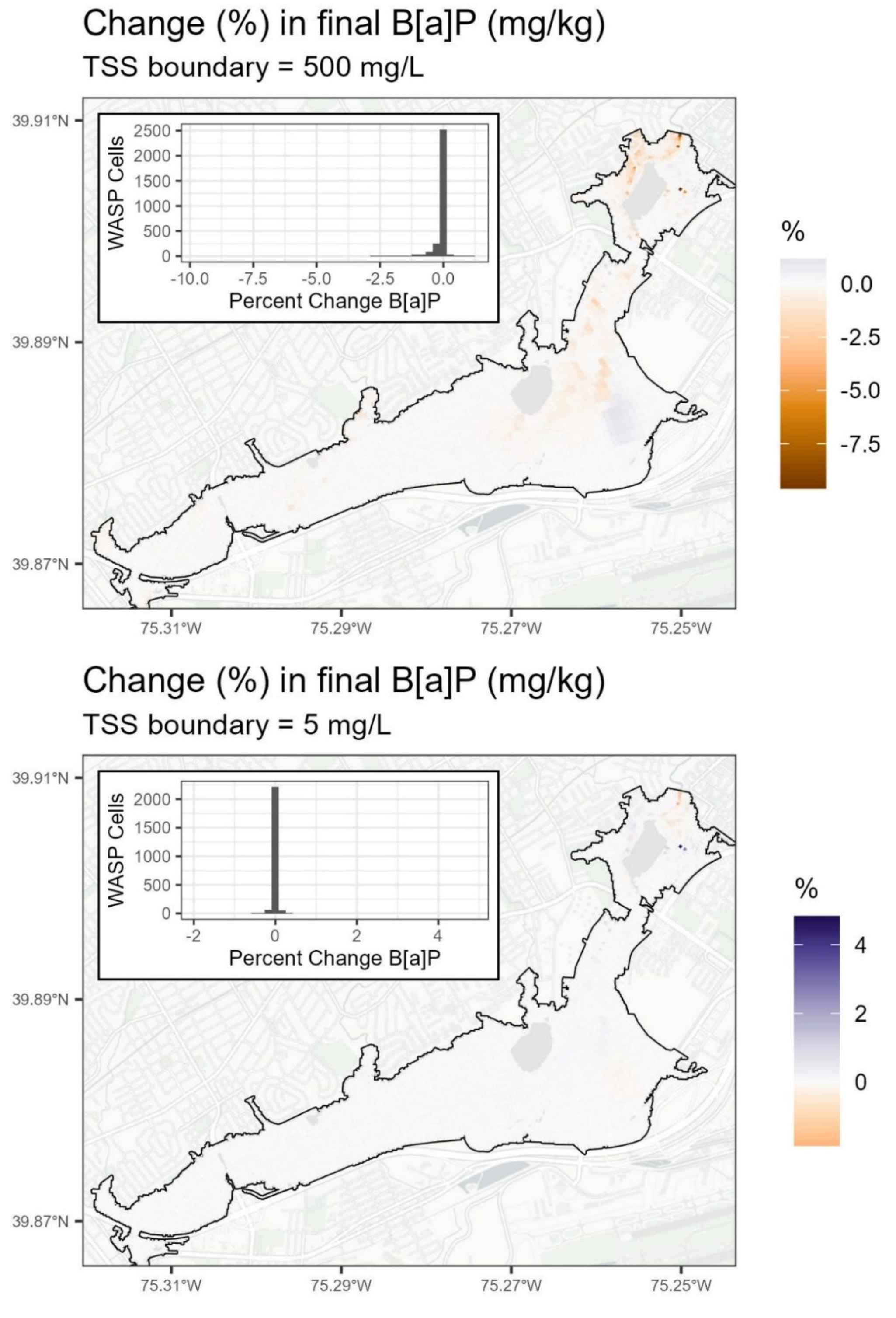

Figure S3. Sensitivity analysis for the TSS boundary condition across the WASP model grid, showing percent difference in B[a]P concentration in sediment at the end of the simulation for TSS boundary = 500 mg/L (top) and TSS boundary = 5 mg/L (bottom) compared to default TSS boundary condition = 50 mg/L. Note differing color scales in top and bottom maps, optimized for display.

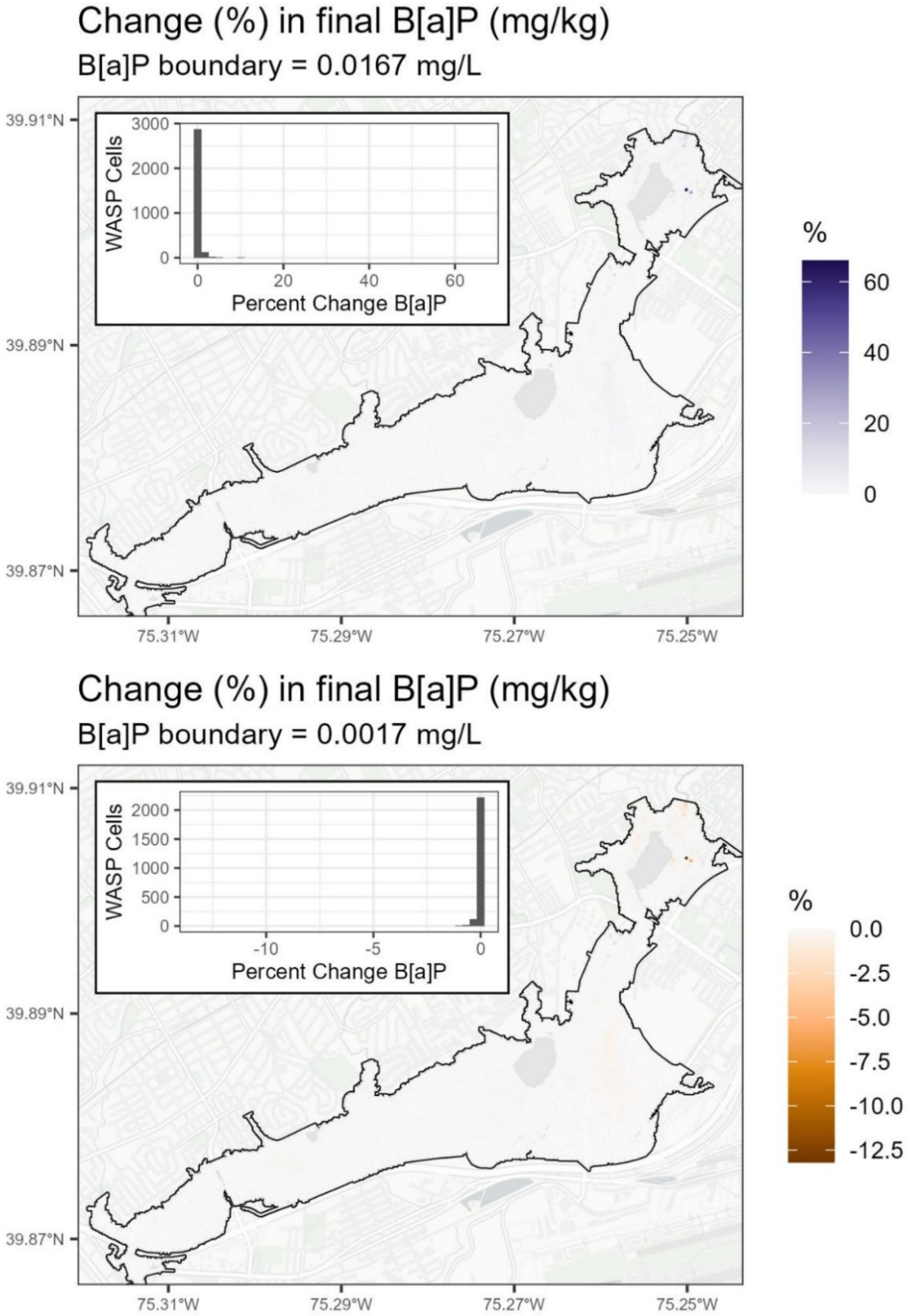

Figure S4. Sensitivity analysis for the B[a]P boundary condition across the WASP model grid, showing percent difference in B[a]P concentration in sediment at the end of the simulation for B[a]P boundary = 0.0167 mg/L (top) and B[a]P boundary = 0.0017 mg/L (bottom) compared to default B[a]P boundary condition = 0.0835 mg/L. Note differing color scales in top and bottom maps, optimized for display.

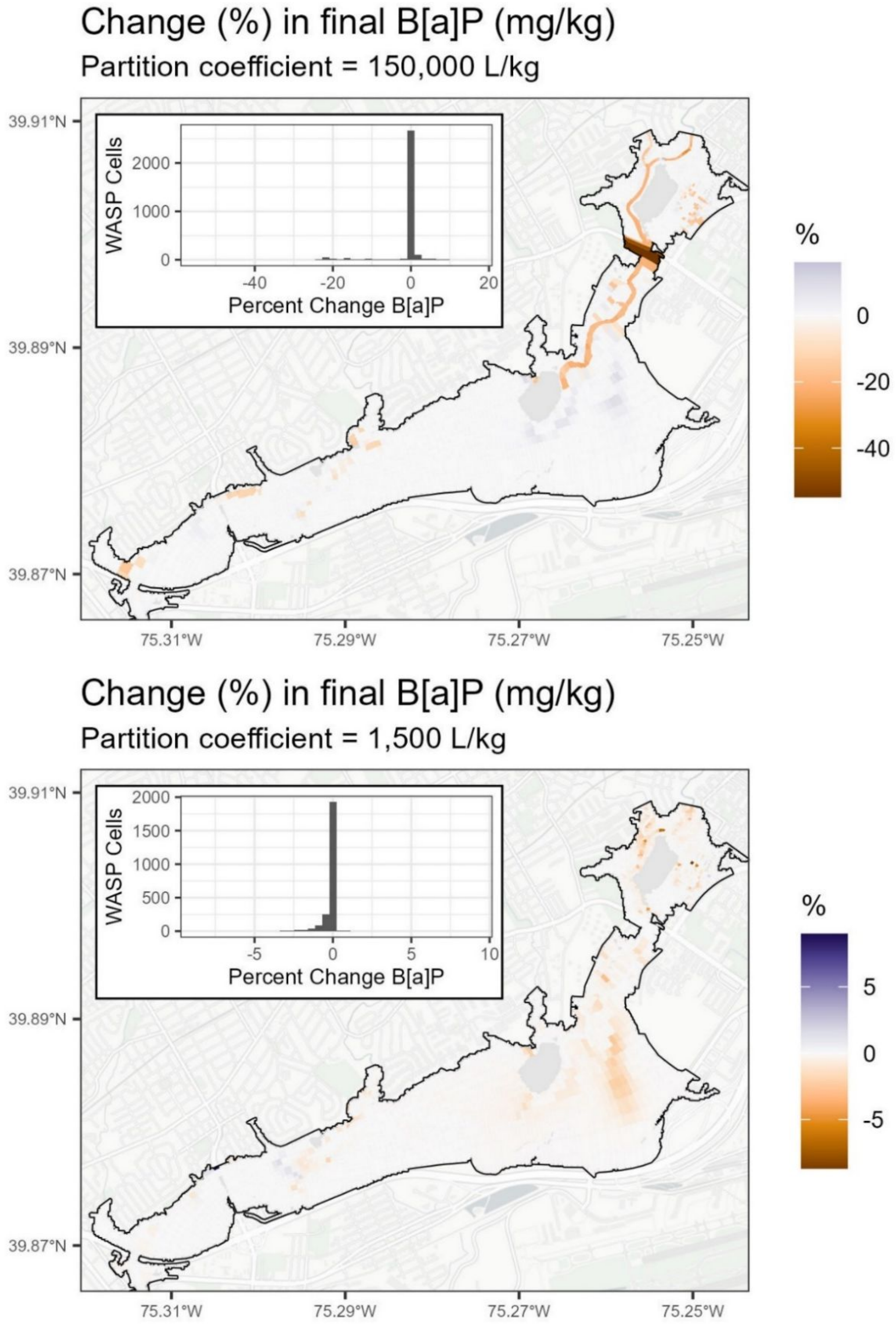

Figure S5. Sensitivity analysis for the partition coefficient (K_d) across the WASP model grid, showing percent difference in B[a]P concentration in sediment at the end of the simulation for K_d = 150,000 L/kg (top) and K_d = 1,500 L/kg (bottom) compared to default K_d = 15,000 L/kg. Note differing color scales in top and bottom maps, optimized for display.

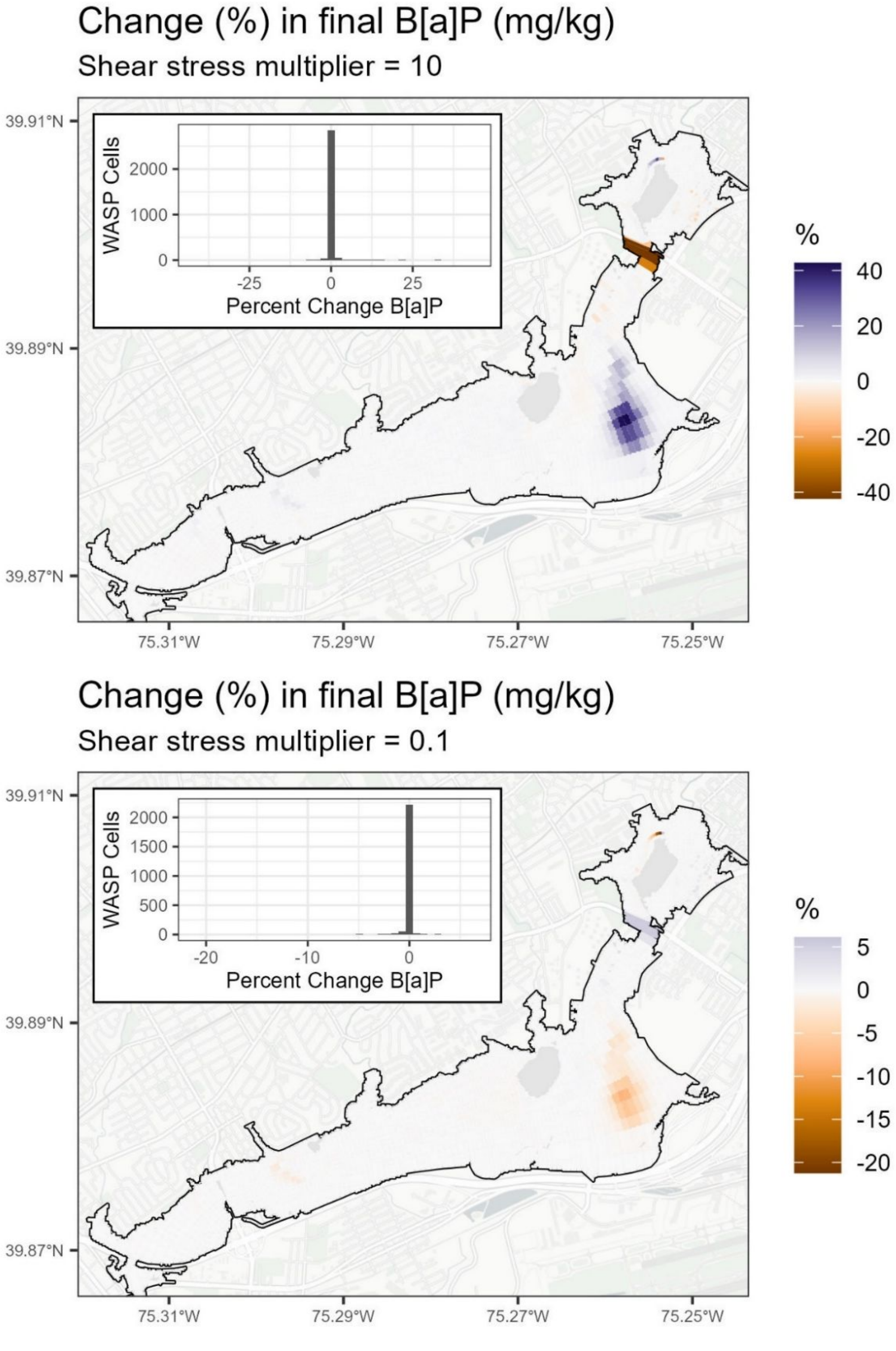

Figure S6. Sensitivity analysis for the shear stress multiplier across the WASP model grid, showing percent difference in B[a]P concentration in sediment at the end of the simulation for shear stress multiplier = 10 mg/L (top) and shear stress multiplier = 0.1 mg/L (bottom) compared to default shear stress multiplier = 1 mg/L. Note differing color scales in top and bottom maps, optimized for display.

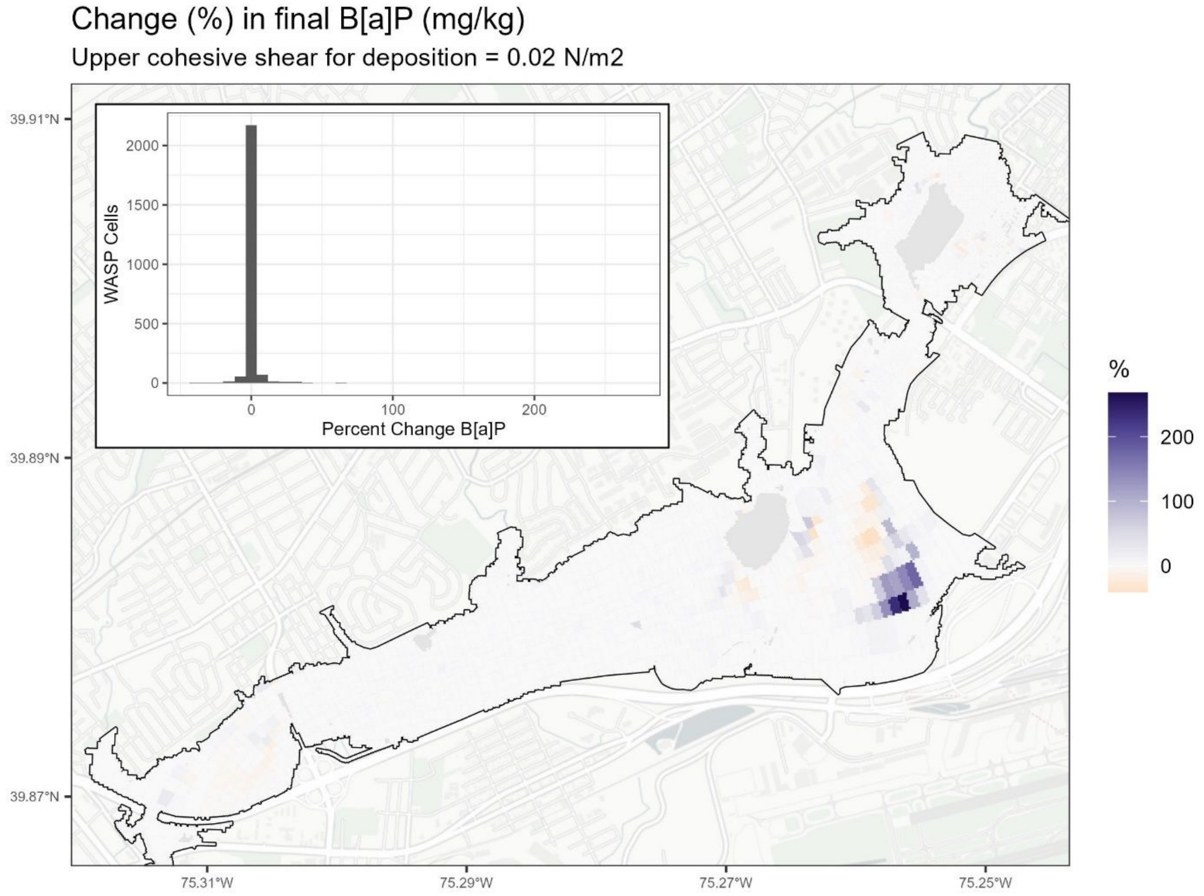

Figure S7. Sensitivity analysis for the upper cohesive shear for deposition across the WASP model grid, showing percent difference in B[a]P concentration in sediment at the end of the simulation for upper cohesive shear for deposition = 0.02 N/m^2 compared to default upper cohesive shear for deposition = 0.2 N/m^2. Note: the default value is the upper range for the parameter, so only a lower value simulation was conducted for sensitivity.

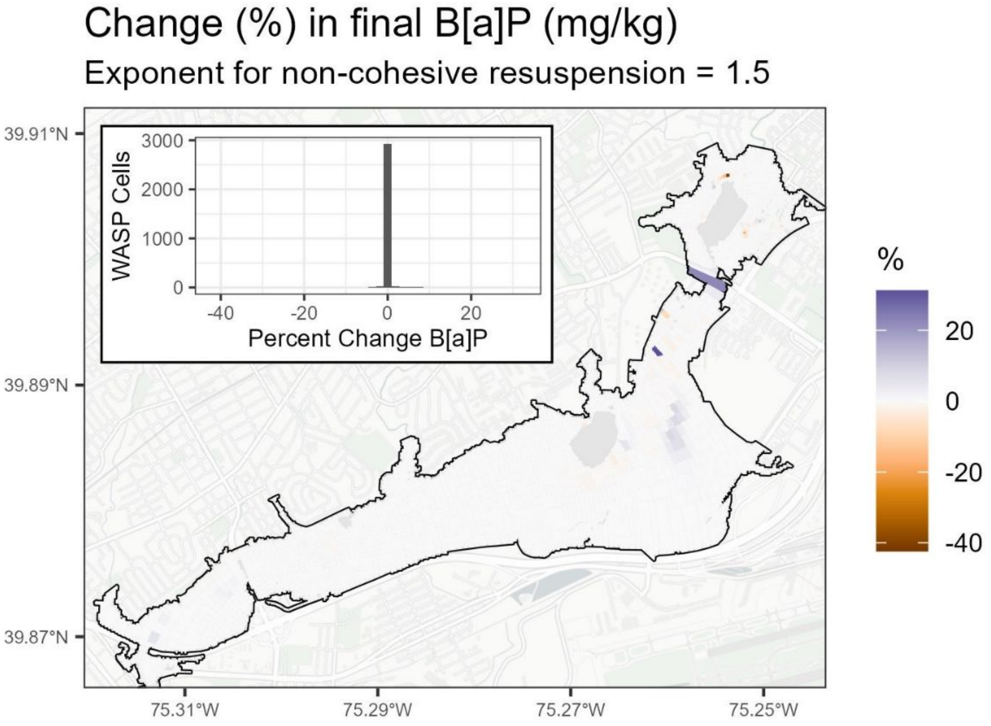

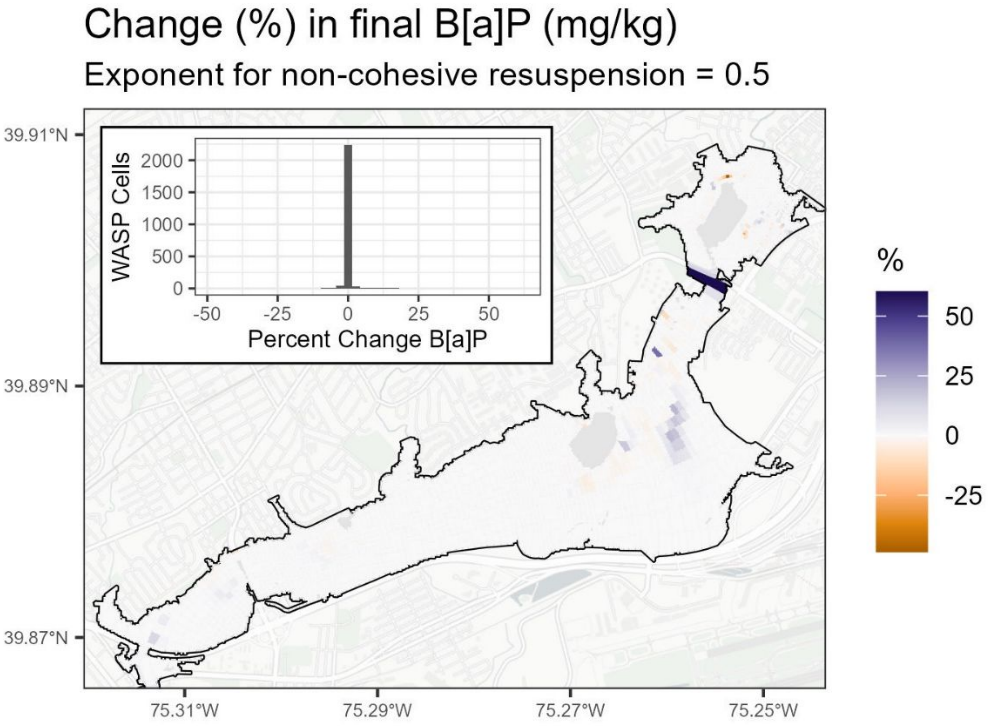

Figure S8. Sensitivity analysis for the exponent for non-cohesive resuspension across the WASP model grid, showing percent difference in B[a]P concentration in sediment at the end of the simulation for exponent for non-cohesive resuspension = 1.5 (top) and exponent for non-cohesive resuspension = 0.5 (bottom) compared to default exponent for non-cohesive resuspension = 1. Note differing color scales in top and bottom maps, optimized for display.

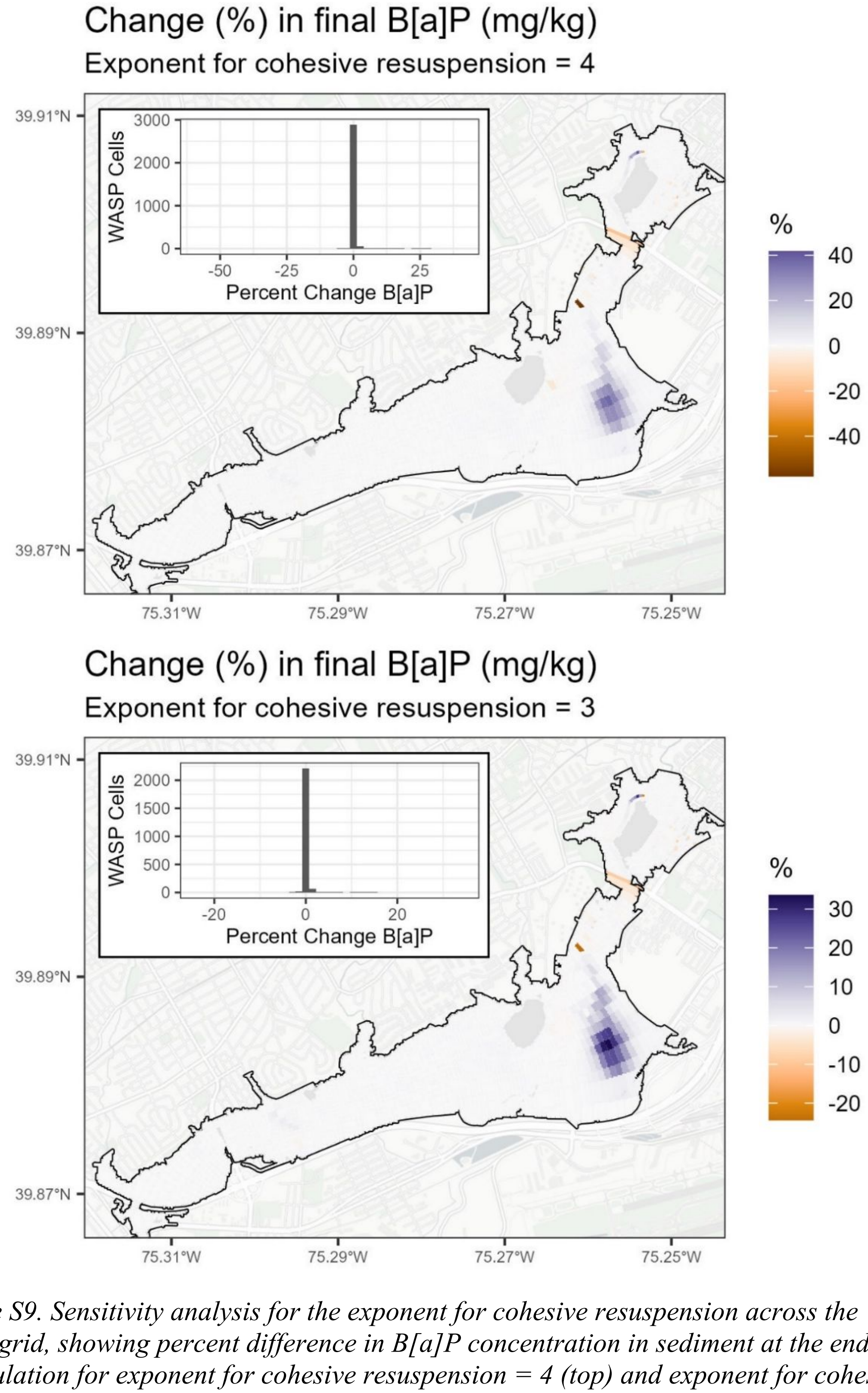

Figure S9. Sensitivity analysis for the exponent for cohesive resuspension across the WASP model grid, showing percent difference in B[a]P concentration in sediment at the end of the simulation for exponent for cohesive resuspension = 4 (top) and exponent for cohesive resuspension = 3 (bottom) compared to default exponent for cohesive resuspension = 1. Note differing color scales in top and bottom maps, optimized for display.

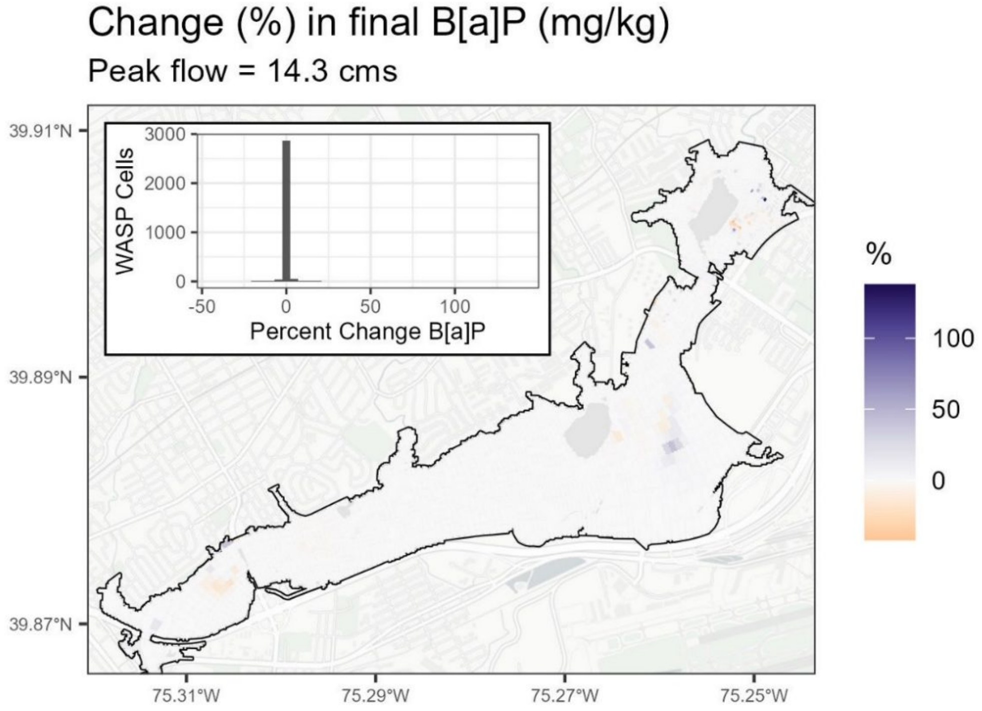

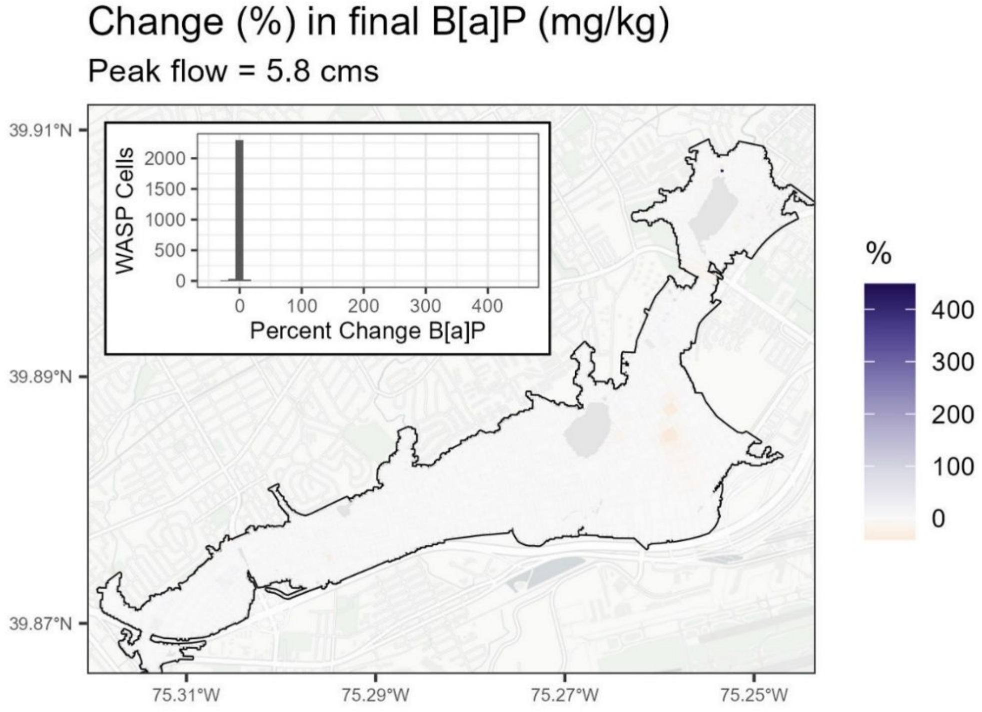

Figure S10. Sensitivity analysis for the peak flow boundary condition across the WASP model grid, showing percent difference in B[a]P concentration in sediment at the end of the simulation for peak flow boundary = 14.3 cms (500-year flood discharge) (top) and peak flow boundary = 5.8 cms (10-year flood discharge) (bottom) compared to default peak flow boundary = 10.8 cms (100-year flood discharge). Note differing color scales in top and bottom maps, optimized for display.

5 REFERENCES

Ambrose, R.B. Jr., B. Avant, Y. Han, and C.D. Knightes. 2017. Water Quality Assessment Simulation Program (WASP8): Upgrades to the Advanced Toxicant Module for Simulating Dissolved Chemicals, Nanomaterials, and Solids. EPA/600/R-17/326. U.S. Environmental Protection Agency, Office of Research and Development, National Exposure Research Laboratory.

Ambrose, R.B. Jr, and T.A. Wool. 2017. WASP8 Stream Transport Model Theory and User's Guide. U.S. Environmental Protection Agency, Office of Research and Development, Washington, DC.

EA Engineering, Science, and Technology, Inc. 2012. Final Sampling and Analysis Plan (SAP)/Field Sampling Plan (FSP) for Remedial Investigation/Feasibility Study (RI/FS) Oversight. Lower Darby Creek Area Superfund Site – Operable Unit 2, Folcroft Landfill. Philadelphia and Delaware Counties, Pennsylvania.

Engineering ToolBox, (2010). Dirt and Mud - Densities . [online] Available at: https://www.engineeringtoolbox.com/dirt-mud-densities-d_1727.html [Accessed 03 06 2023].

Golder. 2018. Revised Final Remedial Investigation Report, Folcroft Landfill and Annex Site, Folcroft, Pennsylvania. Prepared for U.S. Environmental Protection Agency, Philadelphia, PA by Golder Associates, Mt. Laurel, NJ.

Sweet, W.V., Kopp, R.E., Weaver, C.P., Obeysekera, J., Horton, R., Thieler, E.R., and Zervas, C. 2017. Global and regional sea level rise scenarios for the United States. NOAA Technical Report NOS CO-OPS 083. National Oceanic and Atmospheric Administration, Silver Spring, Maryland.

Tetra Tech NUS. 2011. Final Remedial Investigation Report, Remedial Investigation and Feasibility Study (RI/FS), Lower Darby Creek Area (LDCA) Site, Delaware and Philadelphia Counties, Pennsylvania. Prepared for U. S. Environmental Protection Agency Region III, Philadelphia, PA by Tetra Tech NUS, Inc., Newark, DE.

Tetra Tech. 2018. Hydraulic Modeling Report for Existing and Proposed Conditions – Lower Darby Creek Area Superfund Site Remedial Design for Operable Unit 1. Darby Township and Folcroft Borough, Philadelphia and Delaware Counties, Pennsylvania. Prepared by Tetra Tech for U.S. Environmental Protection Agency Region 3, Philadelphia, Pennsylvania.
